# Photoactivable Surfactant Design for In Situ Stabilization of Hydrogel Bioelectronics

**DOI:** 10.1002/adma.74162

**Published:** 2026-07-22

**Authors:** Seungyeon Lee, Donggyun Lee, Soo‐Hwan Lee, Yuri Lee, Jooyeun Chong, Gunho Chang, Hyunjun Kim, Aejin Kim, Kyung Su Kim, Jahyun Koo, Hyojin Lee, Jiheong Kang

**Affiliations:** ^1^ Department of Chemistry Seoul National University Seoul Republic of Korea; ^2^ Biomaterials Research Center Korea Institute of Science and Technology (KIST) Seoul Republic of Korea; ^3^ Department of Materials Science and Engineering Korea Advanced Institute of Science and Technology (KAIST) Daejeon Republic of Korea; ^4^ School of Biomedical Engineering Korea University Seoul Republic of Korea; ^5^ Interdisciplinary Program in Precision Public Health Korea University Seoul Republic of Korea; ^6^ Division of Biomedical Science and Technology, KIST School Korea University of Science and Technology Seoul Republic of Korea; ^7^ SKKU‐KIST, Department of Integrative Biotechnology College of Biotechnology and Bioengineering Sungkyunkwan University Suwon Gyeonggi Republic of Korea

**Keywords:** adhesion, bioelectronics, covalent bond, delamination, materials science, nanotechnology, surfactant, surface energy, wetting

## Abstract

Conductive polymer hydrogels offer unique advantages for soft, stretchable biointerfaces by combining tissue‐like mechanics with high ionic conductivity. However, their reliable integration with hydrophobic substrates and encapsulants essential for electrical insulation and chemical protection remains a major challenge due to poor wetting and interfacial delamination in aqueous environments. Here, we report a facile strategy for the in situ stabilization of conductive polymer hydrogels on hydrophobic substrates using a photoactivable surfactant (PAS). PAS reduces interfacial surface energy and, upon UV activation, forms covalent bonds with stretchable substrates, thereby yielding strong adhesion and long‐term stability under wet conditions. This molecular design also enables photopatterning of hydrogels without compromising performance. Notably, PAS not only stabilizes and patterns conductive hydrogels in situ but also enhances their mechanical and electrical properties. PAS‐integrated hydrogel biointerfaces exhibit robust operation in aqueous environments and demonstrate reliable in vivo electromyographic (EMG) signal recording, underscoring the potential of this approach for next‐generation implantable and wearable bioelectronics.

## Graphical abstract

Photoactivable surfactant additive enables in situ stabilization of hydrogel bioelectronics.

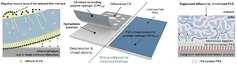

## 1 Introduction

Conductive polymer hydrogels (CPHs) have garnered significant attention as stretchable bioelectrode materials for tissue interfaces due to their chemical and mechanical compatibility with biological tissues. Recent advancements have led to the development of CPHs with enhanced electrical conductivity, tissue‐like modulus, and even intrinsic tissue adhesiveness and stretchability [1, 2, 3]. Among various conductive polymers, PEDOT:PSS has been the most widely used in CPHs owing to its high electrical conductivity, excellent electrochemical stability, and good processability owing to its water dispersibility enabled by PSS doping [4, 5, 6, 7, 8, 9, 10, 11]. However, for stretchable and implantable bioelectronics that must operate reliably under dynamic mechanical deformation and in aqueous biological environments, achieving both mechanical compliance and long‐term device integrity under wet conditions remains a major challenge [12]. Those devices typically require hydrophobic substrates and encapsulation layers to prevent water and ion diffusion, which could disrupt localized neuromodulation or biological signal recording [13, 14, 15]. In contrast, CPHs are inherently hydrophilic and water‐rich, presenting challenges during device fabrication, such as wetting issues and delamination under prolonged exposure to wet conditions.

Two main strategies have been utilized to ensure proper wetting. The first approach involves surface treatments of the substrate, such as oxygen plasma treatment [16], which enhances wettability by introducing hydrophilic functional groups. However, these modifications are often temporary, as they may revert over time due to reorientation of chains from bulk to surface or desorption of the plasma‐induced polar groups, limiting long‐term stability [17, 18, 19]. The second approach incorporates surfactants into the precursor solution. Surfactants effectively lower interfacial energy and improve wetting behavior without requiring additional substrate treatments, simplifying the fabrication process [20, 21]. However, surfactants tend to migrate and diffuse out to the ambient–film interface over the time, leading to interfacial instability, thus limiting their long‐term effectiveness.

Although both approaches temporarily improve wetting, they suffer from weak interfacial interactions, making them vulnerable to water penetration at the interface. This leads to delamination in aqueous environments, limiting their long‐term effectiveness. To achieve durable, long‐term interfacial stability, adhesive interlayers (e.g., epoxy, silane groups, catechol‐based coating) have been proposed [22, 23, 24]. These adhesive layers, which form covalent bonds or physical interactions with both the substrate and hydrogel, significantly enhance adhesion. However, this strategy requires additional fabrication complexities, such as chemical grafting or functionalization. Even chemically treated surfaces, such as those modified with silane‐based reagents, are susceptible to hydrolysis in wet conditions [25], weakening interfacial bonding and limiting device longevity. Furthermore, such interfacial layers are often brittle [26]. Recently, laser treatment has been explored to enhance the adhesion between CPHs and plastic substrates; however, this approach is not applicable to soft elastomeric substrates [27]. Photo‐crosslinkable additives have also been introduced to enable adhesion to elastomeric substrates and high‐resolution patterning; however, their adhesion fundamentally relies on weak physical interactions such as hydrogen bonding, resulting in limited bonding strength [28]. Therefore, an advanced strategy is needed to achieve both wetting and long‐term stability without delamination under wet conditions, even under strain.

In this study, we propose a molecular design of photoactivable surfactants (PAS) as a facile strategy for in situ stabilization of stretchable hydrogel bioelectronic devices. This approach simultaneously satisfies both wetting and wet interfacial adhesion even under strain. The enhancement of adhesion strength by PAS is attributed to two key characteristics: (i) PAS shares structural features with surfactants, effectively reducing the surface energy difference at the interface, and (ii) the photoactivating anchor, benzophenone (BP), covalently bonds with the polymer carbon backbone of the encapsulating material, forming a stable, immobilized network for long‐term bio‐interface stability. Furthermore, the photoactivation of BP moieties enables selective crosslinking in UV‐exposed regions, enabling photopatterning. The resulting PAS‐mixed CPH film (CPH_PAS_) demonstrates high adhesion strength and maintains stable adhesion and conductivity for over 30 days, even in wet environments. To further demonstrate its potential for bioelectronics, biochemical analyses revealed that PAS substantially suppresses immune responses induced by leaching of additives in biological environments, highlighting its ability to ensure long‐term biocompatibility and stable electrochemical performance in vivo. As an electromyogram (EMG) sensor was fabricated using the long‐term stable CPH_PAS_ system, the fabricated EMG sensor enabled stable monitoring of EMG signals from mice for 4 weeks. Additionally, by comparing EMG recordings from brain tumor‐bearing animals with those from healthy controls, we clearly observed a correlation between neural function and movement. Overall, CPH_PAS_ systems present a promising platform for soft and stable bioelectronic devices. Importantly, our approach uniquely integrates stretchability, strong adhesion, wet stability, and patternability‐capabilities that have not been simultaneously achieved in previous PEDOT:PSS hydrogel systems (Table S1).

## 2 Results

### 2.1 In Situ Stabilization of CPHs on Hydrophobic Substrates via PAS

**FIGURE 1:**
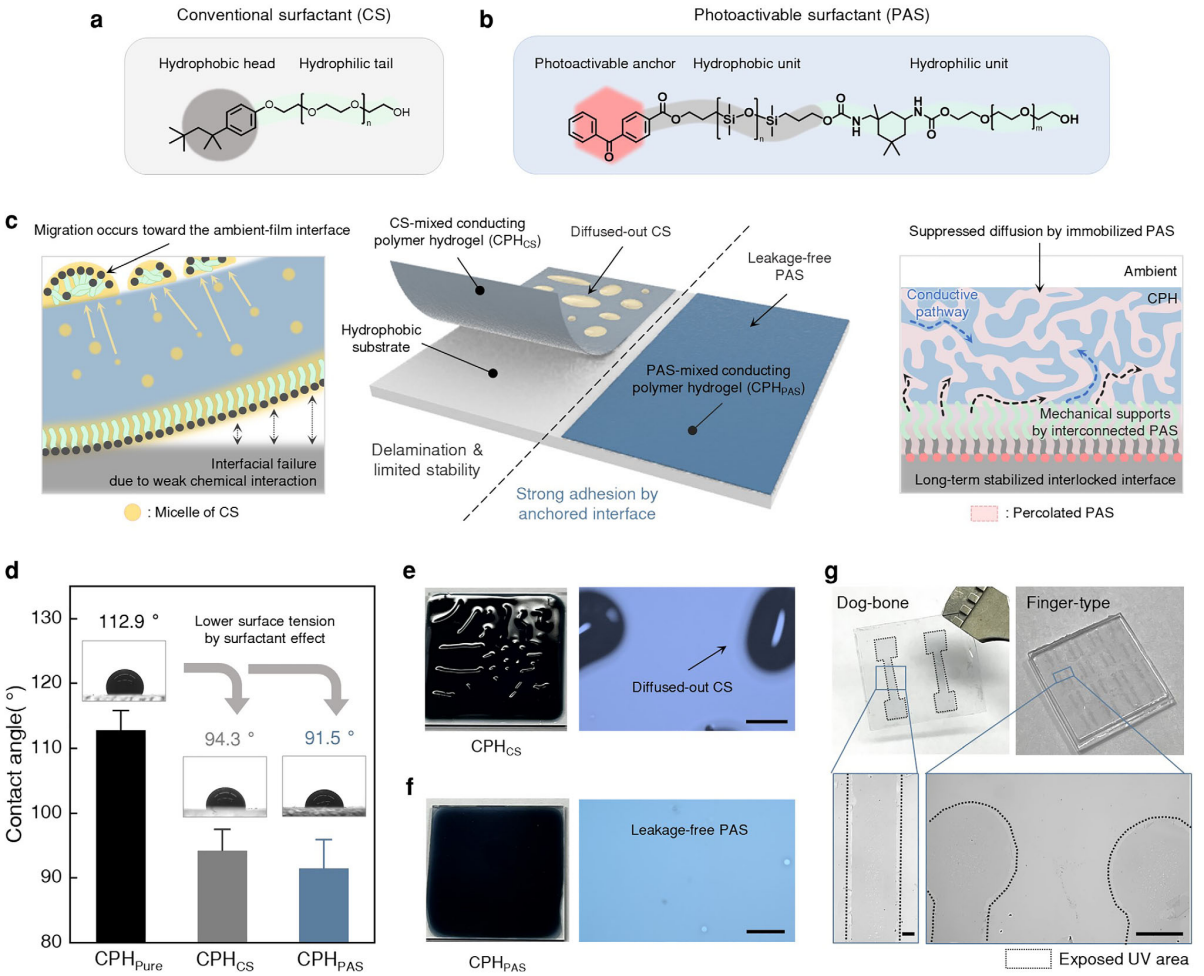
In situ stabilization of conductive polymer hydrogel films using a photoactivatable surfactant (PAS) (a) Molecular structures of a conventional surfactant (CS, Triton X‐100) and (b) the synthesized photoactivable surfactant (PAS), comprising a benzophenone (BP) anchor unit, a hydrophobic polydimethylsiloxane (PDMS) segment, and a hydrophilic polyethylene glycol (PEG) segment. (c) Schematic illustration comparing interfacial behaviors of CPH blended with CS (left) and PAS (right). CS gradually diffuses toward the ambient‐film interface, leading to interfacial failure and delamination. In contrast, PAS enables strong anchoring to the substrate and prevents surfactant migration. (d) Contact angle measurements on PDMS substrates, showing improved wettability upon PAS incorporation (94.3° for the CS‐mixed solution and 91.5° for PAS‐mixed solution). Macroscopic optical images and optical microscopy (OM) images of (e) CPH_CS_ and (f) CPH_PAS_ films. CPH_CS_ exhibit poor uniformity with surface defects with surfactant migration into the surrounding regions. In contrast, CPH_PAS_ show smooth and continuous coverage and PAS remains confined without noticeable diffusion. Scale bar = 500 µm. (g) Photopatterning of CPH_PAS_ on PDMS substrates: a dog‐bone pattern (left, top) and its magnified OM image (left, bottom), and multiple finger‐type patterns (right, top) and their corresponding magnified images (right, bottom). Scale bar = 500 µm.

The newly designed PAS features a molecular architecture composed of a hydrophobic polydimethylsiloxane (PDMS) segment and a hydrophilic polyethylene glycol (PEG) segment, resembling the amphiphilic architecture of a conventional surfactant (CS, Triton X‐100) (Figures 1a, b). PDMS was selected as the hydrophobic block due to its ability to interact favorably with hydrophobic substrates, while PEG was incorporated as the hydrophilic block due to its intrinsic ability to interact with conductive polymers through hydrogen bonding and its high affinity for water [29, 30]. To design the surfactant as a water‐soluble emulsifier considering that water serves as the solvent for the conductive polymer, the hydrophilic PEG chain (2000 Da) and hydrophobic PDMS chain (650–800 Da) were selected based on Griffin's hydrophilic–lipophilic balance (HLB) concept [31]. This combination was expected to place PAS within the HLB range typically associated with water‐soluble emulsifiers (8–16). Additionally, BP, a well‐known photoactivable moiety, was conjugated to one end of the molecule so that PAS could form covalent bonds with surrounding materials upon UV irradiation. The detailed synthesis scheme and structural characterization of the synthetic product are depicted in Figures S1 and S2, respectively.

We envision that PAS would stabilize CPH_PAS_ in two complementary steps: (i) in the solution, it enhances wetting by maximizing interfacial contact and interactions with the substrate (primary stabilization effect); and (ii) the photoactivable anchor forms covalent bonds via C–H insertion upon UV light irradiation (365 nm). Because this process only requires the presence of C–H bonds at the substrate surface, strong adhesion can be achieved on hydrophobic materials without additional surface treatment (secondary stabilization effect).

As illustrated in Figure 1c the in situ stabilization effect of PAS compared to CS. As shown in the left panel, the CS‐mixed film (CPH_CS_) is prone to delamination due to interfacial failure caused by weak physical interactions with the hydrophobic substrate, accompanied by gradual CS migration toward the ambient‐film interface. By contrast, the CPH_PAS_ retains strong adhesion and effectively suppresses surfactant diffusion after photoactivated anchoring. To experimentally verify this, we selected a PDMS‐based substrate (SYLGARD 184) as the model system to investigate the effect of PAS. PDMS is a representative hydrophobic substrate, characterized by its inherently low surface energy (∼21 mN m^−1^) [32], which results in poor wettability with most hydrophilic materials.

The primary stabilization effect of PAS was confirmed through contact angle measurements (Figure 1d). When a PAS‐mixed conductive polymer solution was dropped onto a PDMS substrate, the contact angle decreased to a level comparable to that of a CS‐mixed conductive polymer solution. This result indicates that PAS effectively aligns at the interface and improves wetting behavior [33, 34]. Remarkably, during solution preparation, we also observed pronounced foaming in the PAS‐containing formulation, similar to that seen in detergent solutions (Figure S3 and Movie S1). Moreover, spin‐coating a pure conductive polymer (Pure) solution onto PDMS resulted in poor coating, whereas both CS‐mixed conductive polymer solution and PAS‐mixed conductive polymer solution significantly improved coating uniformity (Figure S4). These results confirm that PAS functions effectively as a surfactant by lowering interfacial energy of the conductive polymer solution.

Although the primary stabilization effect is sufficient to improve initial wetting and film uniformity, long‐term interfacial stability remains challenging when only weak physical interactions are present. As shown for CPH_CS_, CS molecules gradually migrate toward the ambient‐film interface during and after film formation (Figure 1e). Furthermore, interfacial alignment of CS at the film‐substrate interface weakens adhesion, resulting in lower adhesion strength than even the surfactant‐free CPH_Pure_ film (Figure S5). In contrast, the photoactivated anchoring mechanism of PAS provides an additional interfacial stabilization. After UV curing, PAS remains immobilized at the interface and no noticeable migration was observed even after one week (Figure 1f). Interestingly, this anchoring behavior also enables photopatterning of CPH_PAS_ into desired patterns on hydrophobic substrates (Figure 1g). To the best of our knowledge, direct photopatterning of conductive hydrogels on PDMS substrates with micro‐scale resolution has not yet been reported.

### 2.2 Mechanical and Electrical Robustness of CPH_PAS_ Under Wet Conditions

**FIGURE 2:**
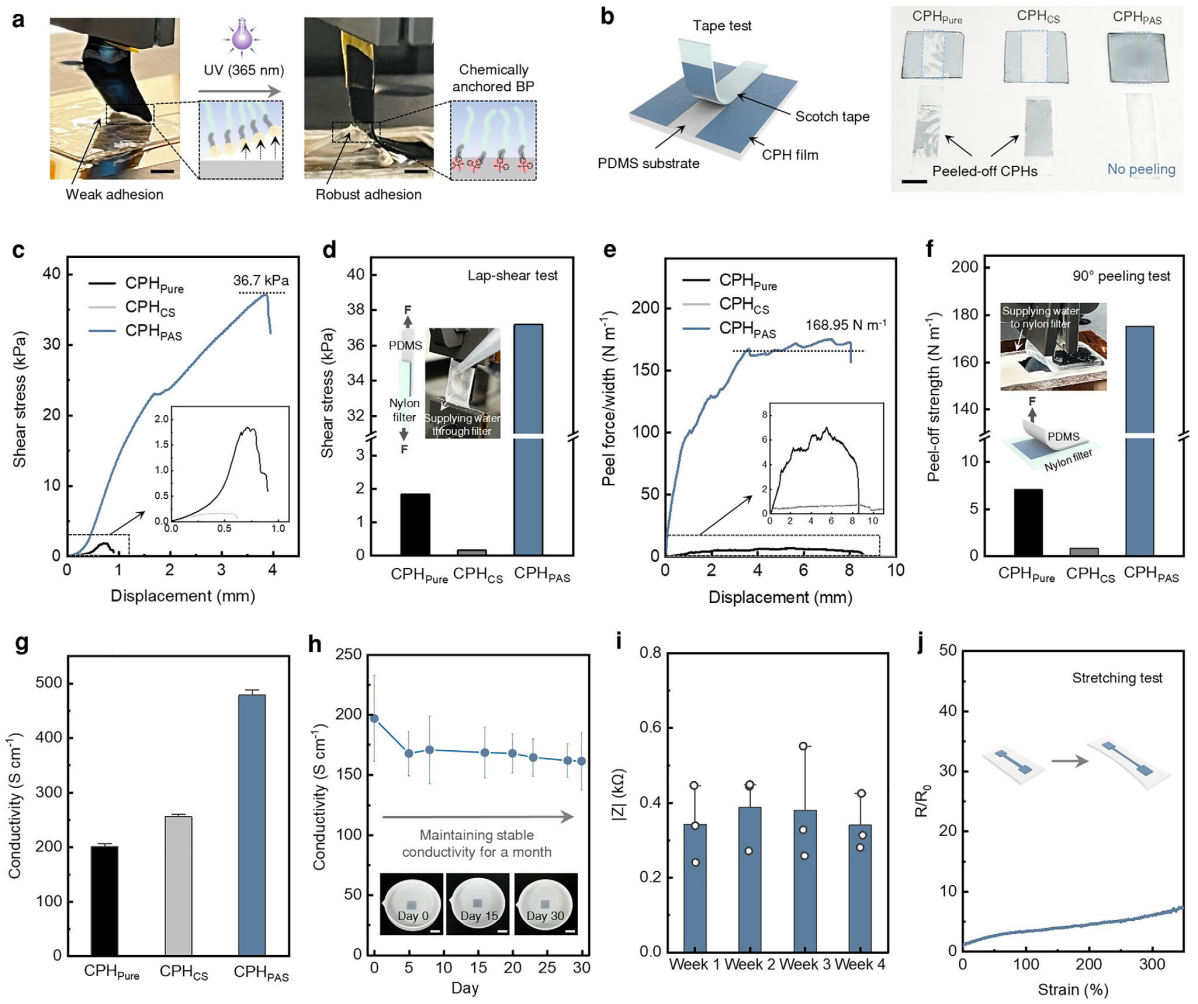
Mechanical and electrical robustness of CPH_PAS_ films under wet conditions (a) Photoactivated adhesion of CPH_PAS_ on PDMS, evidenced by UV‐dependent peeling behavior attributed to anchoring group activation. Scale bar = 5 mm. (b) Scotch tape tests for sub‐micron‐thick films (100–300 nm). CPH_Pure_ and CPH_CS_ films show significant delamination from the PDMS substrates, while the UV‐cured CPH_PAS_ remains stably adhered with no visual signs of peeling. Scale bar = 10 mm. (c) Lap‐shear test under wet conditions using a few‐µm‐thick drop‐cast films. A water‐permeable nylon membrane was used to maintain hydration of the CPH during testing. The CPH_PAS_ exhibits significantly higher shear strength (∼36.7 kPa) compared to CPH_Pure_ (∼2 kPa) and CPH_CS_ (∼0.2 kPa), which delaminate immediately upon loading, and (d) the corresponding shear strength for each sample. (e) 90° peel‐off test under wet conditions using the same film configuration. The CPH_PAS_ exhibits strong interfacial adhesion (∼168.9 N m^−1^), whereas CPH_Pure_ and CPH_CS_ samples show negligible adhesion, and (f) the corresponding peel‐off strength for each sample. (g) Enhanced electrical conductivity of CPH_PAS_ (∼500 S cm^−^^1^), surpassing CPH_Pure_ and CPH_CS_ under identicalpost‐treatment, attributed to PAS‐induced improvement in PEDOT:PSS connectivity. (h) Electrical conductivity of CPH_PAS_ is maintained over 30 days in DI water. After a brief stabilization period (∼2 days), conductivity remains stable, indicating long‐term wet stability. *n* = 3. (Inset: optical images of blue‐colored CPH_PAS_ film on PDMS substrate in DI water at different time points. Scale bar = 10 mm). (i) Electrochemical impedance of CPH_PAS_ at 1 kHz measured over 4 weeks in wet conditions. The impedance remains stable (∼0.4 kΩ), confirming electrochemical stability. *n* = 3. (j) Relative resistance changes of screen‐printed CPH_PAS_ on PDMS under uniaxial strain of up to 350%, showing reliable electrical performance during mechanical deformation.

PAS not only enhances the wetting of the CPH but also significantly improves interfacial adhesion between the CPH and the substrate. We performed peel tests on the drop‐cast CPH_PAS_ film with a thickness of a few micrometers from the PDMS substrate both before and after UV exposure (Figure 2a). In the pristine state, the CPH_PAS_ film detached easily from the PDMS substrate, whereas after UV exposure, it exhibited strong adhesion to PDMS. We hypothesized that this phenomenon was attributed to the photoactivable anchoring effect of BP. When BP was mixed with the PDMS base and exposed to UV light, a quaternary carbon peak appeared at 83 ppm in the ^13^C NMR spectrum (Figure S6), which is consistent with the mechanism in which the carbonyl group of BP generates ketyl radicals that insert into neighboring C–H bonds of PDMS chains, forming covalent linkages with the substrate [35, 36, 37].

To examine whether this adhesion could be maintained in a sub‐µm thin film, a Scotch tape test was performed [38] (Figure 2b and Movie S2). The UV‐treated CPH_PAS_ exhibited no delamination from the PDMS substrate following tape peeling. In contrast, the CPH_Pure_ and CPH_CS_ were easily delaminated, as evidenced by dark blue residues transferred onto the tape side. Electrical resistance measurements further supported this observation. While CPH_Pure_ and CPH_CS_ became insulating immediately after peeling, the CPH_PAS_ exhibited minimal changes in resistance, indicating stable adhesion (Figure S7).

PAS effectively promoted adhesion not only for ultrathin films with submicron thickness, but also for films several micrometers thick. We briefly confirmed that a ∼10 µm‐thick screen‐printed CPH_PAS_ film on PDMSremained stably adhered even under aqueous conditions that typically induce delamination due to expansion forces from swelling of the hydrogel (Figure S8 and Movie S3). To quantitatively assess the interfacial adhesion strength between CPH_PAS_ and PDMS, we conducted two adhesion tests: lap‐shear and 90° peel‐off test. Using drop‐cast films with a thickness of 2∼5 µm, both tests were performed under wet conditions and consistently demonstrated the robust adhesion of CPH_PAS_. A water‐permeable nylon membrane filter was placed on the CPH_PAS_ (Figure 2b, inset). The nylon membrane filter was chosen as backing for its hydrophilic, microporous structure that promotes strong interlocking with PEDOT:PSS hydrogels and enables stable, hydrated, and deformation‐free measurements [12, 27]. In the lap‐shear test, CPH_Pure_ and CPH_CS_ detached immediately upon loading, showing low shear strength (∼ 0.2 kPa), while the CPH_PAS_ exhibited a markedly higher shear strength (∼ 36.7 kPa) (Figure 2c, d). Similarly, in the 90° peel‐off test, where the nylon side was fixed and the PDMS layer peeled off, the CPH_PAS_ showed substantially stronger adhesion (∼ 168.9 N m^−1^), consistent with the lap‐shear results (Figure 2e, f). These results indicate that the PAS‐stabilized conductive polymer network remains mechanically robust against both hydrogel swellingexpansion and externally applied deformation (Figure S9). Additional adhesion test data obtained at different PAS concentrations (*C*_PAS_) are presented in Figure S10.

In addition to its mechanical robustness under wet conditions, CPH_PAS_ provides more efficient charge transport pathways. Notably, CPH_PAS_ exhibited a conductivity of ∼500 S cm^−1^, surpassing both CPH_Pure_ (∼200 S cm^−1^) and CPH_CS_ (∼250 S cm^−1^) (Figure 2g), suggesting enhanced electrical connectivity within the PEDOT network. X‐ray photoelectron spectroscopy (XPS) analysis further supports this result, showing a decrease in the S 2p peak intensity of PSS relative to PEDOT (Figure S11), indicating that the PEG moiety of PAS removes the insulating PSS shell from PEDOT via Coulombic interactions, improving electrical conduction [28].

CPH_PAS_ also demonstrated excellent electrical stability. When thin CPH_PAS_ were immersed in deionized (DI) water and monitored over a month, conductivity remained remarkably stable after an initial stabilization period (up to 2 days), during which any residual PEDOT:PSS was leached out (Figures 2h and S12). In contrast, CPH_CS_ underwent immediate delamination upon immersion (Figure S13). Stable electrochemical performance was also observed, with impedance remaining stable (∼0.4 kΩ at 1 kHz) over four weeks (Figures 2i and S14). Along with its high electrical conductivity and electrochemical stability, the CPH_PAS_ maintained stable electrical performance during mechanical deformation. A screen‐printed, dog‐bone‐shaped CPH_PAS_ showed stable resistance changes (*R*/*R*_0_< 10) under uniaxial stretching up to 300%, indicating its suitability for soft and stretchable bioelectronic interfaces. (Figure 2f). Additional control data for a GOPS (3‐glycidoxypropyltrimethoxysilane)‐based system, in which GOPS is commonly employed as a silane crosslinker to promote adhesion to PDMS substrates, are shown in Figures S15 and S16. Notably, these results highlight a simple yet highly effective strategy for constructing soft, stretchable hydrogel biointerfaces with robust adhesion to hydrophobic substrates.

### 2.3 Photopatterning Mechanism Based on the Formation of a Percolated PAS Network

**FIGURE 3:**
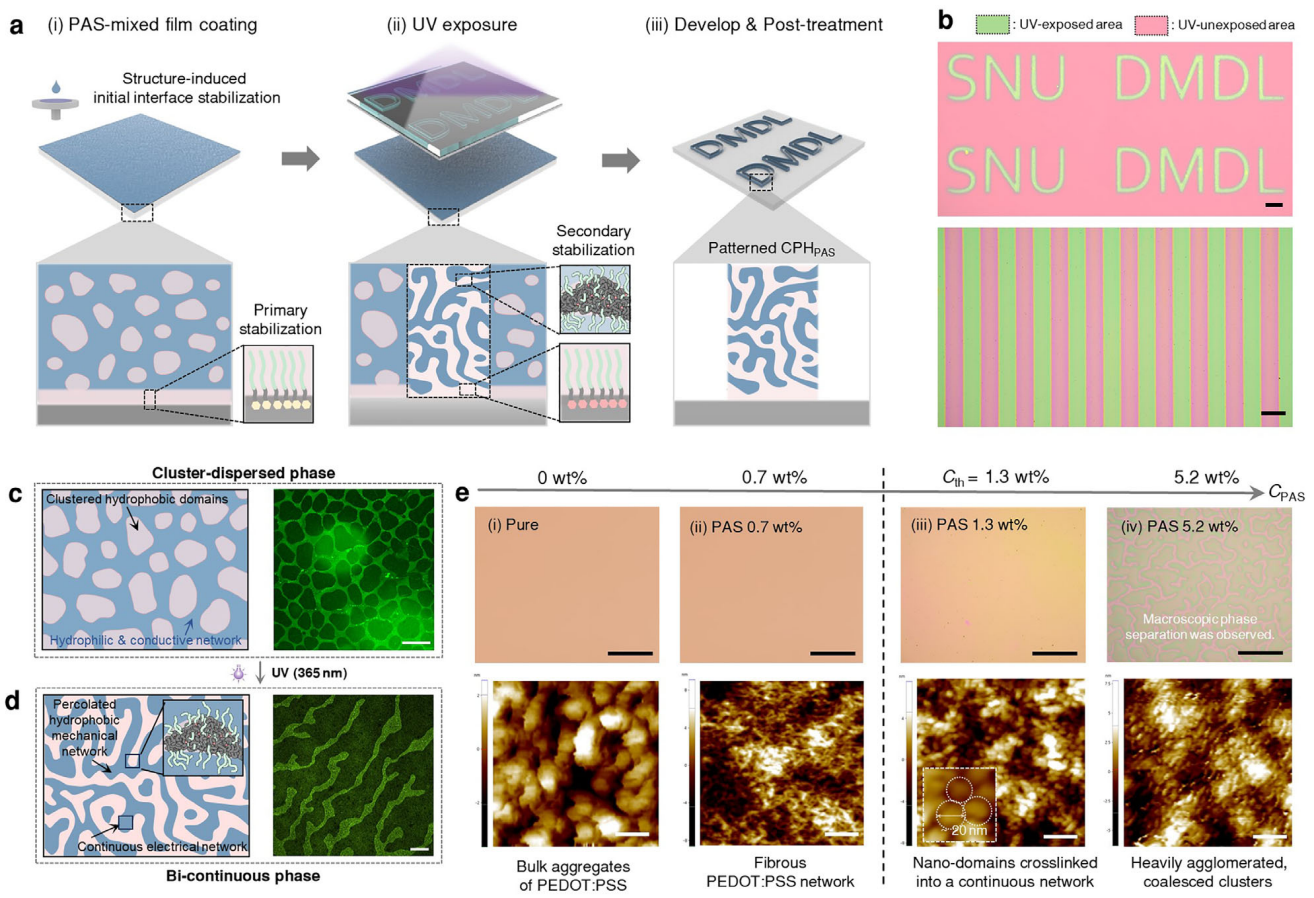
Photopatterning mechanism based on the formation of a percolated PAS network. (a) Schematic illustration of the photopatterning process using PAS‐induced stabilization effects. (i) A PAS‐mixed conductive polymer solution is spin‐coated onto a plasma‐treated substrate, forming a uniform film with primary stabilization effects. (ii) Upon UV exposure (365 nm), photoactivating anchors of PAS are activated in selective exposed regions, inducing covalent bonding with the substrate and promoting percolation among PAS. (iii) During the development process, the UV‐unexposed regions of the film are removed, followed by treatment with polar solvents to enhance conductive pathways. (b) Photopatterned CPH_PAS_ exhibiting fine microscale resolution. UV‐lithographic patterning enables high‐fidelity structures, including alphabetic characters with a width of 30 µm (top) and line pattern with a 70 µm pitch (bottom). Scale bar = 100 µm. (c‐d) Schematic diagrams and confocal fluorescence microscopy images describing the bicontinuous structure driven by percolation of PAS and UV activation. (c) Schematic illustration of dispersed PAS‐derived hydrophobic domains within the CPHPAS matrix prior to UV irradiation (left), and the corresponding confocal microscopy image showing isolated aggregated domains in the hydrophilic matrix (right). Scale bar = 20 µm. (d) Schematic illustration of the interconnected, bicontinuous network formed in CPH_PAS_ upon UV exposure (left), and the corresponding confocal microscopy image of CPH_PAS_ after UV irradiation (right). In the confocal images, the light green color corresponds to dye‐containing hydrophilic domains, whereas the dark regions represent dye‐excluded hydrophobic domains. Scale bar = 100 µm. (e) Optical microscopy and AFM images of CPH_PAS_ prepared with various PAS concentrations: (i) Pure PEDOT:PSS, (ii) 0.07 wt%, (iii) 1.3 wt% (threshold concentration, *C*_th_), and (iv) 5.2 wt% in PEDOT:PSS solution. Unlike films below the *C*_th_, films above *C*_th_ exhibit a crosslinked PAS network formed through percolation and BP‐mediated crosslinking. Scale bar = 50 µm (OM images), 100 nm (AFM images). The use of the ‘SNU’ logo in this figure is authorized by Seoul National University.

When the CPH_PAS_ samples were immersed in water to assess the stability in aqueous environments, we found that only the UV‐exposed regions remained intact while the unexposed regions did not. Based on this observation, we hypothesized that photopatterning would be feasible, which was subsequently confirmed (Figure 1g). A PAS‐mixed conductive polymer solution was spin‐coated onto a plasma‐treated substrate to form a thin film (100 – 300 nm) (Figure 3ai). Upon 365 nm UV irradiation for 10 min, BP was selectively activated at the exposed sites. The activated BP groups anchor PAS to the substrate while simultaneously linking neighboring PAS molecules within the matrix, resulting in the formation of a crosslinked network (Figure 3aii). Once formed, this interconnected network remains insoluble while retaining water, thereby behaving as a hydrogel structure. The film is then immersed in a developer solution (IPA:D.I. water = 2:3) with gentle agitation, dissolving the unexposed regions and retaining the UV‐cured regions on the substrate, thus forming the desired pattern (Figure 3aiii). Even after development, PAS in the exposed areas remained intact. To maximize electrical performance, the resulting CPH_PAS_ films were immersed in DMSO for 10 s and then dried at 100°C for 1 h to promote the formation of enhanced conductive pathways [4]. This optimized lithography process enables relatively high‐resolution photopatterning of CPH_PAS_, as observed in lettering patterns with a minimum width of 30 µm (Figure 3b). Confocal microscopy revealed that prior to UV irradiation, hydrophobic PDMS‐derived domains exist as aggregated clusters dispersed within the hydrophilic PEDOT:PSS matrix (Figure 3c). Because these domains were spatially disconnected, they were unable to maintain structural integrity in aqueous environments. However, during UV exposure, covalent anchoring proceeds while chain mobility is enhanced by the accompanying photothermal effect, enabling molecular rearrangement. As a result, the initially isolated hydrophobic domains become interconnected through BP‐mediated crosslinking, producing a mechanically continuous network. Consequently, the initially phase‐separated structure evolves into an interconnected, bicontinuous architecture in which hydrophobic domains become linked throughout the hydrophilic matrix (Figure 3d). This interconnected structure effectively suppresses excessive swelling and improves hydrogel stability, explaining why the patterned regions remain intact after development. The formation of the bicontinuous network was further supported by AFM images (Figure S17).

We defined this structure a hydrogel for the following reasons. (i) Pure PAS forms a crosslinked hydrogel upon UV irradiation, as evidenced by its transition toward more solid‐like viscoelastic behavior in frequency sweep measurements after UV exposure (Figures S18 and S20). (ii) CPH_PAS_ samples above the critical concentration remained structurally intact and exhibited noticeable swelling after UV irradiation even without DMSO treatment, indicating that PAS‐induced crosslinking alone is sufficient to generate a water‐stable hydrogel network independent of PEDOT:PSS connectivity (Figure S19 and Movie S6). Since DMSO is known to improve the connectivity between PEDOT:PSS chains [39, 40], excluding DMSO reduces additional reinforcement from PEDOT:PSS and allows the hydrogel formation to be attributed primarily to the PAS network. (iii) CPH_PAS_ above the threshold concentration displays an elastic plateau (G′ > G″) within the linear viscoelastic region and shows reversible macroscopic elasticity under cyclic loading (Figures S21 and S22). Overall, these findings support the formation of mechanically interconnected and percolated networks with a bicontinuous morphology.

We also observed significant morphological difference in the PAS network across different PAS concentrations (*C*_PAS_). When *C*_PAS_ was 0.7 wt%, 1.3 wt%, and 5.2 wt% of PEDOT:PSS solution, photopatterning was only successful above a certain threshold concentration (*C*_th_ = 1.3 wt%) (Figure S23). This behavior suggests that network formation is strongly dependent on PAS concentration. AFM analysis further revealed distinct morphological differences below and above *C*_th_ (Figure 3e). CPH_Pure_ with DMSO post‐treatment showed large spherical aggregates with an insulating PSS shell surrounding the PEDOT (Figure 3ei), as previously reported [40]. At concentrations below the *C*_th_, PAS acted in a manner analogous to a secondary dopant [41], inducing a fibrous morphology through modulation of PEDOT–PSS interactions, removal of excess PSS shells, and enhancement of π–π stacking between PEDOT chains (Figure 3eii). The enhanced conductivity compared to the CPH_Pure_ film further supports the role of PAS (Figure 2g). However, as the concentration increases, the higher local fraction of PAS promotes percolation among PAS domains, thereby leading to the formation of clusters. At the *C*_th_, where inter‐cluster connectivity emerges, UV activation drives these domains to be subsequently crosslinked into a continuous network via benzophenone (BP) anchors (Figure S24). AFM image consistently showed that when *C*_PAS_ exceeds the *C*_th_ (*C*_PAS_ > 1.3 wt%), these nano‐sized domains organize into a continuous, interconnected network (Figure 3e iii). These domains are considerably smaller than those observed in either pure PEDOT or pure PAS systems. Dynamic light scattering (DLS) analysis also confirmed this observation. Mixing PAS with PEDOT:PSS generated significantly smaller particles (20–50 nm) (Figure S25), suggesting the formation of initial PAS nano‐domains within CPH_PAS_. To investigate the morphology within the bulk hydrogels, we analyzed our pre‐hydrogel samples using small‐angle X‐ray scattering (SAXS) (Figure S26). Once the PAS concentration exceeds the *C*_th_, the absolute slope in the low‐q region (∼0.02 Å^−^^1^) increases, indicating stronger scattering intensity. These results collectively suggest that PAS is largely dispersed as isolated entities below *C*_th_ but forms an increasingly interconnected network above the threshold concentration. Based on these results, we concluded that above *C*_th_, PAS assembled into a bicontinuous network within the PEDOT:PSS matrix, where interconnected PAS domains provided mechanical continuity while PEDOT:PSS maintained electrical conductivity. As a result, no delamination was observed, even in films ranging from several tens of nanometers to several tens of micrometers in thickness (Figure S8). However, when PAS is incorporated at excessively high concentrations (*C*_PAS_ > 2.6 wt%), the percolation‐driven assembly becomes overly enlarged, leading to the formation of large aggregates that hinder electrical conduction (Figures 3e iv and S27). Considering its combination of mechanical robustness, electrical stability, and lithographic processability, CPH_PAS_ represents a promising material platform for implantable bioelectronic applications.

### 2.4 Evaluation of In Vivo Biocompatibility of CPH_PAS_

**FIGURE 4:**
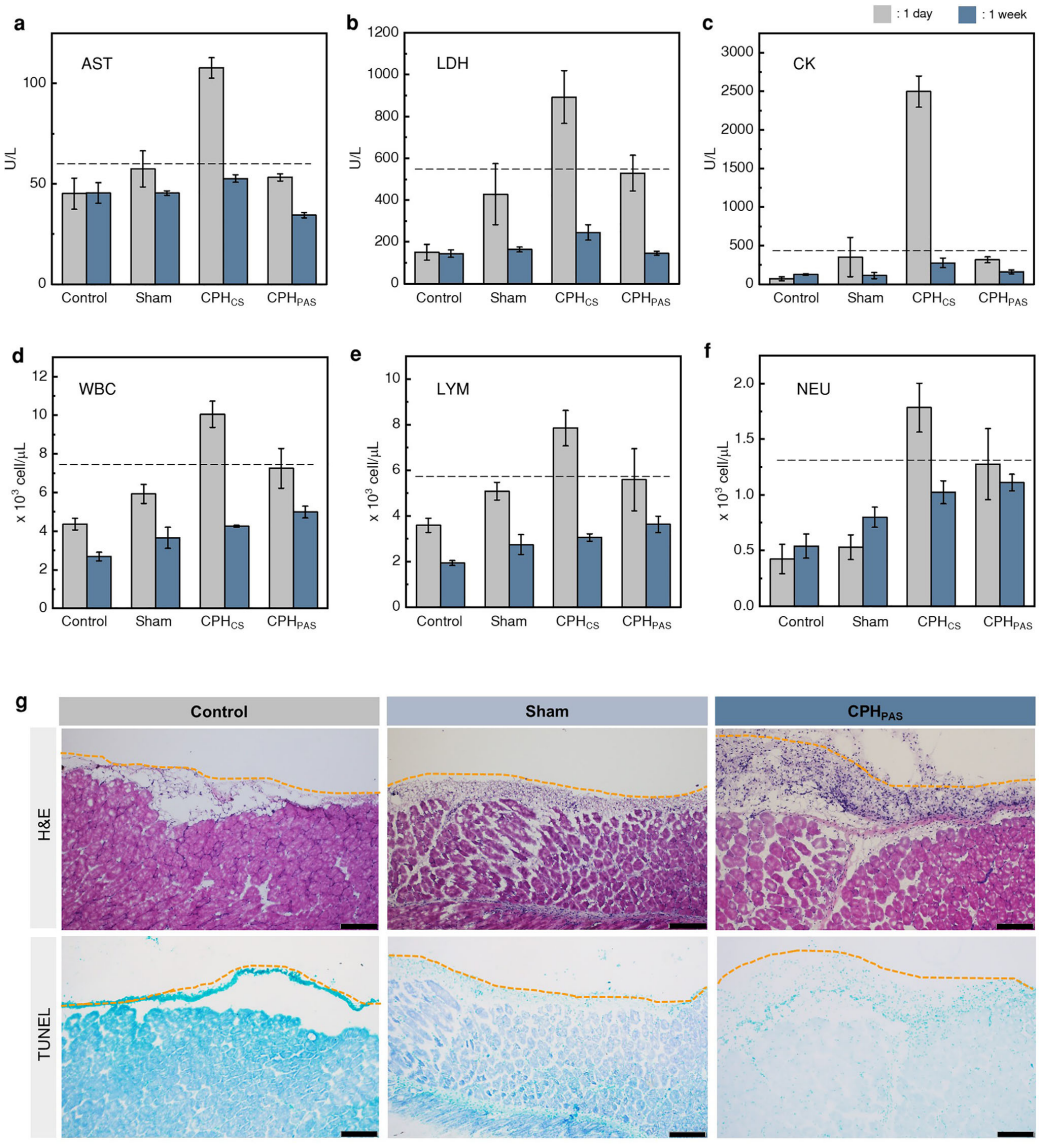
Biocompatibility of CPH_PAS_. (a–c) Serum biochemical analysis of liver, kidney, and muscle function markers at the indicated time points. (a) Levels of aspartate aminotransferase (AST), (b) lactate dehydrogenase (LDH), and (c) creatine kinase (CK) (*n*  =  3). (d–f) Complete blood count (CBC) analysis at 1 day and 1week post‐implantation of each material (PDMS, CPH_CS_, CPH_PAS_). (d) Quantitative changes in total white blood cell (WBC) counts and (e) differential counts of lymphocytes (LYM) and (f) neutrophils (NEU) were measured in blood samples at each time point (*n* = 3). In the figure (a–f), the dashed line represents the normal reference range for mice. Data are presented as mean values ± SEM. (g) Histological analysis of muscle tissue 1 week post‐implantation. Representative images of hematoxylin and eosin (H&E; upper) and TUNEL staining (lower). The orange dashed line indicates the location of the implant. Scale bar = 200 µm.

Biocompatibility is a critical criterion for materials intended for in vivo applications. To evaluate the biocompatibility of CPH_PAS_, hematological analysis of blood toxicity markers at 1 day and 1 week post‐implantation was conducted to examine whether the PAS additive induces any biological response, particularly those associated with additive leaching. CPH_CS_ was included as a comparison group expected to exhibit additive‐leaching‐related toxicity. The analyses revealed no significant changes in either the Sham (implanted with PDMS only) or CPH_PAS_ groups (see Figure S28c for details of the surgical procedure and the extracted muscle area used for cell staining). In contrast, mice receiving CPH_CS_ implants exhibited acute hematological toxicity and muscle toxicity at the early time points (Figure 4a‐c). CS has also been shown to induce liver toxicity, consistent with previous reports [42]. As shown in Figure 1e, CS gradually leaches out over time, whichlikely accounts for the cause of its observed toxicity. Moreover, compared with the CPH_CS_, CPH_PAS_ elicited a negligible foreign body response in vivo, indicating superior suitability for implantation (Figure 4d–f). The distinct difference in biocompatibility between the two samples clearly indicates that the choice of electrode additives can significantly influence not only the operational stability of the electrode but also its in vivo biocompatibility. The other serum biochemical factors and CBC measurement results were comparable across groups (Figures S29 and S30). In short, the absence of pathological responses in CPH_PAS_ supports the conclusion that the introduction of PAS does not induce additional biocompatibility concerns relative to established biocompatible PEDOT:PSS‐based materials.

To assess the in vivo biocompatibility of the materials, Sham, CPH_CS_, or CPH_PAS_ implants were placed into the right thigh muscle of mice, sutured, and left in place for one week. Histology of muscle tissue prepared to enhance visualizing the layered structure revealed that the CPH_PAS_ group exhibited no apparent abnormalities on hematoxylin & eosin (H&E) or TUNEL staining (Figure 4g). Notably, no abnormal findings were observed in the tissue layer above the CPH_PAS_ implant, as indicated by the upper orange dashed line. In comparison, foreign body–like cells were predominantly observed in the superficial muscle layer above the implant site (orange dashed line) in the CPH_CS_ group (Figure S31a), and TUNEL staining showed positive apoptotic signals within the muscle layer (Figure S31c). Consistently, skin histology revealed that foreign body–like response in the CPH_CS_ group (Figure S31b, d), whereas the CPH_PAS_ group exhibited no apparent abnormalities on H&E or TUNEL staining (Figure S32). Based on these one week findings in skin and longitudinally sectioned muscle, we next performed a more comprehensive histological assessment of muscle tissue at both 1 and 4 weeks post‐implantation to evaluate short‐ and long‐term tissue responses.

Histological examinations using H&E and Masson's trichrome staining revealed no significant disparities in muscle morphology between the treated and non‐treated control mice at either time point (Figure S33). Furthermore, to assess apoptosis induced by material implantation, TUNEL staining demonstrated no significant difference in apoptotic cells between CPH_PAS_‐implanted muscle and either untreated controls or Sham (PDMS implants) (Figure S34), indicating that CPH_PAS_ exhibits equivalent in vivo biocompatibility. Taken together, these findings indicate that CPH_PAS_ demonstrates excellent in vivo biocompatibility comparable to that of the Sham and untreated controls, eliciting minimal inflammatory or apoptotic responses in surrounding muscle tissue.

### 2.5 In Vivo Operational Stability of CPH_PAS_ as Bioelectronic Interfaces

**FIGURE 5:**
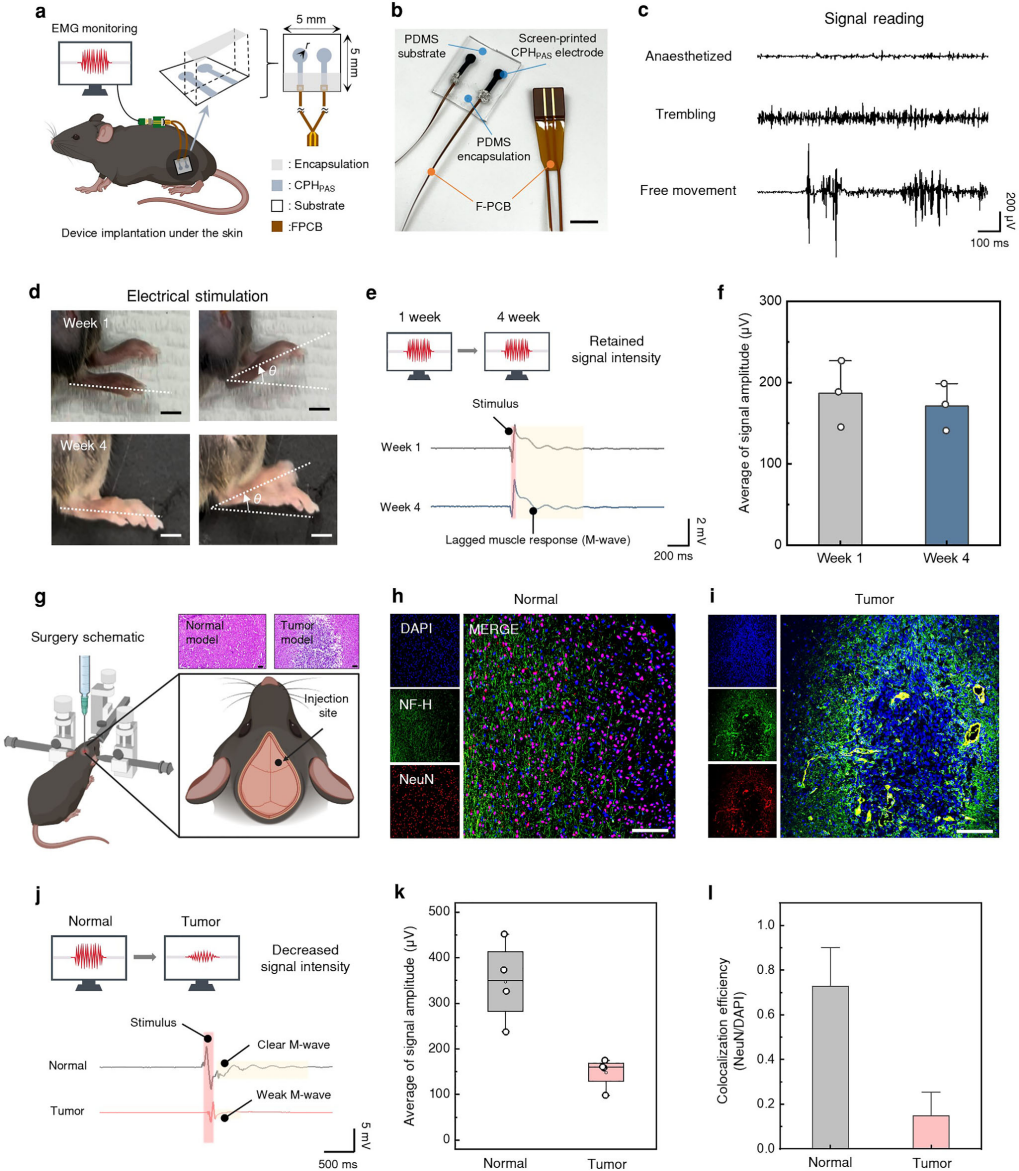
In vivo operational stability of CPH_PAS_ as bioelectronic interfaces (a) Experimental setup for electromyography (EMG) with the implantable hydrogel probes. (b) Implantable conductive hydrogel probes are fabricated by screen printing with 1 mm line width and 1 mm radius of contact pad encapsulated with a thin PDMS layer. F‐PCB is assembled onto each electrode with silver paste. Scale bar = 5 mm (c) EMG signal recordings from the mouse leg muscle under varying experimental conditions. (d‐f) Long‐term durable functionality tests of hydrogel sensor devices during 4 weeks. (d) Leg movement upon electrical stimulation using the CPH_PAS_ hydrogel‐based sensor at week 1 and week 4 post‐implantation. The implanted hydrogel electrodes maintained stable performance and effective bioelectronic interfacing over the 4‐week period. Scale bar = 5 mm. (e) Comparable amplitudes of EMG signals at week 1 and week 4 after implantation. (f) Averaged signal amplitudes under muscle stimulation at week 1 and week 4 (average of 10 signals each, *n* = 3). Only a slight decrease was observed, demonstrating stable in vivo functionality over 4 weeks. (g) Schematic image of stereotaxic surgery for the brain tumor mouse model. (Top inset: representative H&E image of normal mouse model and tumor mouse model. Scale bar: 100 µm). (h,i) Fluorescence microscopy image of brain tissue stained for nuclear (DAPI), neuronal filament (NF‐H), and neuronal nuclei (NeuN) of (h) normal mouse and (i) tumor mouse model. Scale bar = 100 µm. (j) Representative EMG traces between normal and tumor‐bearing models. Tumor‐bearing mice showed significantly reduced signals compared to normal model. (k) Quantitative comparison of EMG amplitude between normal and tumor‐bearing mouse models (*n* = 4) by averaging ten signals. Data are presented as mean values ± SEM. Statistical analyses were performed by two‐sided Student's t tests. *p* < 0.01. (l) Quantification of colocalization efficiency between NeuN and DAPI‐stained nuclei (*n* = 3). Data are presented as mean values ± SEM. Statistical analyses were performed by two‐sided Student's t tests. *p* < 0.01. Schematic images were created with BioRender.com.

To validate the in vivo functional performance of the sensor device, we utilized CPH_PAS_ as hydrogel electrodes, demonstrating excellent performance and long‐term stability for both electromyogram (EMG) stimulation and recording. We fabricated implantable conductive hydrogel probes by screen printing methods with a 1 mm line width and a 1 mm radius of contact pad (Figure 5a). A soft PDMS substrate was used for conformal contact with tissue, and flexible printed circuit board (F‐PCB) was assembled onto the hydrogel probes to connect to signal acquisition system. The entire device was subsequently encapsulated with a thin spin‐coated PDMS layer (Figure 5b). The device was implanted into the right thigh muscle of each mouse. EMG recordings were obtained while anesthetized, awake, and during free movement. Distinct EMG signal patterns were observed across each condition, indicating that the hydrogel‐based sensor could reliably monitor muscle activity in vivo (Figure 5c and Movie S4). Control experiments conducted without electrical stimulation verified that the recorded signals originated from physiological muscle activity rather than filtering artifacts (Figure S35 and Movie S7). To confirm the performance of CPH_PAS_ hydrogel probes, we compared their signal‐to‐noise ratio (SNR) with that of CPH_GOPS_‐based control devices. As shown in Figure S36, representative EMG traces obtained under electrical stimulation indicate that CPH_GOPS_ exhibits a noticeably higher noise floor and a lower SNR compared to CPH_PAS_. Consistent with the impedance trends (Figure S16c), the relatively limited swelling of GOPS‐crosslinked films [4] might constrain ionic transport within the film, contributing to reduced EMG signal fidelity. The mechanical robustness, electrical stability, and biocompatibility of CPH_PAS_ also enabled long‐term signal acquisition of EMG signals after 4 weeks of implantation. Hydrogel probes were implanted in healthy mice, and the initial EMG signal was recorded immediately after implantation and re‐measured four weeks later. The probes exhibited consistent functionality throughout the implantation period, without any significant loss of signal acquisition capability. Muscle stimulation was successfully achieved in both week 1 and week 4 (Figure 5d and Movie S5), suggesting that the electrode remained stable in vivo over the period. TheEMG signals recorded after stimulation at week 1 and week 4 showed minimal variation in waveform and amplitude, confirming the ability to obtain consistent signals over time (Figure 5e). Quantitative analysis of ten independent recordings further showed only a modest reduction in average signal amplitude after four weeks (Figure 5f), supporting the long‐term stability of the implanted sensors under physiological conditions.

Overall, these findings demonstrate that the fabricated hydrogel probes reliably detect in vivo muscle activity and effectively discriminates between healthy and brain tumor‐bearing mice. We used a murine brain tumor model to compare EMG signals of the right thigh muscle between healthy control and tumor‐bearing mice (Figure 5g). After confirmation of glioblastoma formation in the brain (Figure 5G, top insets), we performed immunohistochemical staining for NeuN, a marker of neuronal nuclei frequently used to assess neuronal integrity, and neurofilament heavy chain (NF‐H). In brain tumor‐bearing mice, we observed markedly reduced NeuN immunoreactivity along with co‐aggregation of NeuN and NF‐H at the tumor margin (Figures 5h, i), suggesting neuronal damage and potential disruption of both structural and functional connectivity within affected regions. Representative EMG traces (Figure 5j) and quantitative analysis (Figure 5k) revealed that tumor‐bearing mice exhibited a significantly lower average EMG amplitude compared to the control group. The marked decrease in NeuN‐positive nuclei within the tumor model, as shown in quantitative colocalization of DAPI and NeuN (Figure 5l), directly correlates with the reduction of EMG amplitude (Figure 5k). Based on the organization of the ascending sensory pathway (from peripheral nerves to the cortex) [43] and reduction of NeuN, we propose that observed neuronal disruption in the brain hinders signal transmission. This is consistent with previous reports that decreased NeuN is commonly associated with functional impairments [44, 45, 46]. Linking the neuronal loss and structural disruption to the observed electrophysiological impairment, this EMG‐based evaluation mayprovide a strategy for identifying muscle atrophy associated with brain tumors.

## 3 Conclusion

By addressing the leaching of surfactant‐type additives in conductive hydrogels, PAS provides a promising material for processability and long‐term operation of bioelectronic interfaces. Upon incorporation of PAS, resulting CPHs are stabilized through two steps: reducing the interfacial surface energy with hydrophobic substrates, and thenfollowed by immobilization through photoactivable anchors. These in situ stabilization effects of PAS facilitate both anchoring to the substrates and percolation throughout the polymer matrix, enabling the photopatterning of CPH_PAS,_ therby providing a significant advantage for device fabrication. This work may offer a new design strategy for developing stable, patternable, and biocompatible conductive hydrogels tailored for advanced bioelectronic interface applications.

## Author Contributions

S.L., D.L., S.‐H.L., H.L., and J. Kang designed the concept and experiments. S.L. and Y.L. synthesized and characterized PAS. S.L., D.L., G.C., and J. Kang developed the methods for photopatterning and sensor fabrication. S.L., D.L., and J.C. conducted the mechanical and electrical characterizations. S.L. and G.C. obtained AFM data. S.L., D.L., Y.L., J.C., G.C., H.K., and J. Kang analyzed the data. S.‐H.L., A.K., and H.L. designed the animal studies and carried out the in vivo experiments. S.‐H.L., and A.K. performed biochemical and histological analysis, established and conducted experiments using normal model and murine brain tumor‐bearing mice. K.S.K. set up the electromyogram measurement system, obtained and analyzed the electromyogram measurements, and was advised by J. Koo. J. Kang supervised the overall project. All authors discussed the results and jointly wrote the manuscript.

## Conflicts of Interest

The authors declare no conflicts of interest.

## Supporting information




**Supporting File 1**: adma74162‐sup‐0001‐SuppMat.docx.

**Supporting File 2**: adma74162‐sup‐0002‐MovieS1.mp4.

**Supporting File 3**: adma74162‐sup‐0003‐MovieS2.mp4.

**Supporting File 4**: adma74162‐sup‐0004‐MovieS3.mp4.

**Supporting File 5**: adma74162‐sup‐0005‐MovieS4.mp4.

**Supporting File 6**: adma74162‐sup‐0006‐MovieS5.mp4.

**Supporting File 7**: adma74162‐sup‐0007‐MovieS6.mp4.

**Supporting File 8**: adma74162‐sup‐0008‐MovieS7.mp4.

## Data Availability

The data that support the findings of this study are available in the supplementary material of this article.
