# Supplementary material for: Photoactivable Surfactant Design for In Situ Stabilization of Hydrogel Bioelectronics
Source: Adv Mater. 2026 Jul 22;38(49):e74162. doi: 10.1002/adma.74162 (PMC13532466; doi:10.1002/adma.74162)
Supplement: Supplementary file 1 [file ADMA-38-e74162-s004.docx]

Supplementary Materials for

**Photoactivable surfactant design for in-situ stabilization of hydrogel bioelectronics**

Seungyeon Lee *et al.*

*Corresponding author : Jiheong Kang, [jiheongkang@snu.ac.kr](mailto:jiheongkang@snu.ac.kr)

**This PDF file includes:**

Materials and Methods

Figs. S1 to S37

Table S1

References

**Other Supplementary Materials for this manuscript include the following:**

Movies S1 to S7

**Materials and Methods**

**Materials**

4-Benzoylbenzoic acid (99%), N-ethyl carbodiimide hydrochloride (EDC·HCl), 4-(dimethylamino)pyridine (DMAP) (99%), isophorone diisocyanate (98%), acetone (99.5%), and chloroform (99.5%) were purchased from TCI (Japan). Poly (ethylene glycol) (M_n_ = 2000 g mol−1), dibutyltin dilaurate (DBTDL) (95%), dichloromethane (anhydrous, 99%), tetrahydrofuran (anhydrous, 99%), dimethyl sulfoxide (DMSO) (99%), triethylamine (99.5%), 2′,4′,5′,7′-tetrabromofluorescein (Eosin Y) and magnesium sulfate were purchased from Sigma-Aldrich (USA). Hydroxyl terminated PDMS (Mn = 600-850 g mol^−1^) was purchased from Gelest (USA). PEDOT:PSS aqueous solution (Clevios PH1000, 1.0–1.3% solid content) was purchased from Heraeus Electronic Materials. All materials were used without purification. Sylgard 184 base and catalyst were purchased from the Dow Chemical Company (USA). Silver paste (ELCOAT, P-100) was purchased from CANS. Note that in all fabrication processes using PEDOT:PSS in this study, a pre-filtering step was performed using a PTFE filter (pore size of 0.45 µm) purchased from Millex to remove PEDOT:PSS aggregation.

**Synthesis of PAS (BP-PDMS-PEG)**

**(1) BP-PDMS-OH**

A total of 50 mL of anhydrous DCM was added to a flask containing HO-PDMS-OH (13.73 g, M.W. = 600–850, 18.94 mmol, 1 eq) and DMAP (1.157 g, 9.47 mmol, 0.5 eq) under argon atmosphere. In another flask, 100 mL of anhydrous DCM was added to 4-Benzoylbenzoic acid (BP, 3 g, 13.26 mmol, 0.7 eq) and EDC∙HCl (5.45 g, 28.42 mmol, 1.5 eq) under argon atmosphere. After stirring for 1 h, the mixture solution of BP, EDC∙HCl was added dropwise to the mixture of HO-PDMS-OH, DMAP. The resulting mixture was stirred for overnight at room temperature in a light-blocked state. After reaction, the solution was extracted three times with 1 M HCl to remove catalysts and by-products of EDC reagent, and then the lower DCM layer was collected. The following reaction mixture was dried over MgSO4, filtered and concentrated under vacuum to get the crude product. To obtain a product BP-PDMS-OH, where BP is attached to only one side of PDMS, further purification was conducted by silica column chromatography using 2% acetone in chloroform as the eluent.

**(2) BP-PDMS-PEG**

Isophorone diisocyanate (1.786 mL, 8.43 mmol, 1 eq), DBTDL (0.1 mL, 0.168 mmol, 0.02 eq) were dissolved in anhydrous THF (10 mL) under argon atmosphere. BP-PDMS-OH (9.27 g, 1 eq) dissolved in anhydrous THF (90 mL) was added dropwise. The mixture was stirred for overnight at room temperature to synthesize intermediate BP-PDMS-IU product. Then, poly(ethylene glycol) (PEG, 18.54 g, M.W. = 2000, 9.27 mmol, 1.1 eq), dried under vacuum overnight at 80°C, was dissolved in anhydrous THF (50 mL) and DBTDL (0.1 mL, 0.168 mmol, 0.02 eq) was added. BP-PDMS-IU solution was added dropwise to the PEG bath under argon atmosphere, and then the mixture was stirred for overnight at room temperature. After the reaction, the following mixture was concentrated under vacuum. To obtain a final product, BP-PDMS-PEG, silica column chromatography was conducted using 5% triethylamine in acetone as the eluent. All processes are carried out with as little light as possible.

**NMR Spectroscopy**

^1^H and ^13^C NMR spectroscopy were recorded on a Bruker AVANCE NEO spectrometer (500 MHz). All test samples were prepared in deuterated chloroform at room temperature. Chemical shifts are referenced to the residual solvent signal.

**Photopatterning of PAS Film**

For photopatterning, PDMS was mainly used as the substrate, with wafers utilized for fine pattern definition. PAS-mixed PEDOT:PSS solution was prepared by mixing 1 mL PEDOT:PSS solution (Clevios PH1000) with 0.013 g of PAS (for PAS 1.3 wt% solution, at a PEDOT:PSS-to-PAS weight ratio of 1:1) or 0.026 g of PAS (for PAS 2.6 wt% solution, at a PEDOT:PSS solute-to-PAS weight ratio of 1:2). To ensure complete mixing of PAS, the mixture was vortexed for 5 min, sonicated for 10 min, and then vigorously stirred overnight using a magnetic stirrer. The resulting stock solution was degassed prior to film fabrication to remove any trapped air bubbles. CPH_PAS_ solution (PAS 1.3 wt% or 2.6 wt% in PEDOT:PSS) was spin-coated onto a UV plasma-treated substrate at 3000 rpm for 60 s with ramp 10 s to form an approximately 150 nm-thick film. The resulting CPH_PAS_ thin film was irradiated with UV light (365 nm, 40 W) for 10 min through a photomask. And then the film was subsequently immersed in a developer solution (IPA:deionized water = 2:3) with gentle agitation, dissolving the unexposed regions while retaining the UV-cured patterns on the substrate. After development, samples were dipped in DMSO for 10 s and annealed at 100°C for 1 h. Final samples were soaked in deionized water bath to wash out residual additives.

**Fabrication of Electromyogram (EMG) Sensors**

Electromyogram (EMG) sensor was fabricated by screen sameprinting CPH_PAS_ ink onto UV plasma-treated PDMS substrates with screen printing masks. CPH_PAS_ ink (PEDOT:PSS solute: PAS = 1:1 or 1:2 weight ratio) was fabricated with reference to the following paper [3]: 0.16 g of PAS was dissolved in 2.67 mL of aqueous DMSO solution (water:DMSO = 85:15 v/v) and stirred overnight. The pure PEDOT:PSS solution was frozen in liquid nitrogen bath for 10 min. The frozen PEDOT:PSS was lyophilized for 72 h to remove water and leave only solid PEDOT:PSS. 0.08 g of lyophilized PEDOT:PSS (for 1:1, 0.04 g for 1:2 weight ratio of PEDOT:PSS-to-PAS) were dissolved in mixture of above PAS solution, followed by grinding and overnight stirring. Masks for screen printing were made by cutting a polyethylene terephthalate (PET) film with a CO2 laser cutter (Trotec). After the CPH_PAS_ was printed, the substrate was flipped over and irradiated with 365 nm UV light for 30 min toward the transparent substrate-side. As with the photopatterning process, the samples were immersed in DMSO, annealed at 100°C for 1 h, and then soaked in D.I. water for washing and swelling. Customized Polyimide/Au flexible printed circuit boards (F-PCB) were attached on each electrode line with silver paste, and then dried at 60°C for 2 h. After verifying the electrical connection, the electrodes were encapsulated by coating PDMS with curing agents.

**Contact Angle Measurements**

Contact angle analysis was done by free interface droplet analysis equipment (SmartDrop, FEMTOBIOMED). A 2 µL drop of each conducting polymer solution was very slowly and carefully contacted and removed from the bare PDMS substrate (base:curing agent = 20:1) using a pipette and measured immediately. The contact angle was automatically calculated and derived by the program.

**Dynamic Light Scattering (DLS) Analysis**

The hydrodynamic diameters of the pure PAS, pure PEDOT:PSS, and mixed PAS-PEDOT:PSS solution were measured by dynamic light scattering (DLS) using a laser diffraction particle size analyzer (MasterSizer 3000, Malvern). Each sample, pure PEDOT:PSS, pure PAS, and PAS-mixed PEDOT:PSS solution (PAS 2.6 wt% of PEDOT:PSS) was prepared by diluting 20 µL of the original solution in 8 mL of D.I. water to obtain a nearly transparent solution.

**Small-Angle X-ray Scattering (SAXS) Measurements**

Small-angle x-ray scattering (SAXS) measurements were performed at the 4 C SAXS II beamline at the Pohang Accelerator Laboratory (PAL) in Pohang, Republic of Korea. Storage ring energy was 3.0 GeV with an energy resolution (ΔE/E) of 2 × 10−4. The sample-to-detector distances were set at 4 m, 1 m, and 20 cm. For solution SAXS measurements, PAS-mixed PEDOT:PSS solutions were prepared by mixing 1 mL of PEDOT:PSS solution with 0.007 g, 0.013 g, 0.026 g, and 0.052 g of PAS to obtain concentrations of 0.7 wt%, 1.3 wt%, 2.6 wt%, and 5.2 wt%, respectively. To ensure complete mixing of PAS, the mixture was vortexed for 5 min, sonicated for 10 min, and then vigorously stirred overnight using a magnetic stirrer. For bulk film SAXS measurements, the samples were prepared by dropping each solution onto a Teflon plate containing a 1 × 1 cm PDMS mold, followed by drying overnight. The film thickness was adjusted to approximately 50 µm. The resulting films were irradiated with UV light (365 nm, 40 W) for 1 h, dipped in DMSO for 10 s and then annealed at 100°C for 1 hr. Bulk film SAXS data were measured with fully dried films.

**Atomic Force Microscopy**

AFM images were obtained using atomic force microscope (NX10, Park Systems) with the tip of PR-A160TS.

**Confocal Fluorescence Microscopy**

Confocal fluorescence microscopy was performed to characterize the morphological features of the bicontinuous structure in CPH_PAS_. For sample preparation, a PAS 2.6 wt% in PEDOT:PSS solution was mixed with 0.1 wt% Eosin Y (2′,4′,5′,7′-tetrabromofluorescein) and stirred overnight to ensure homogeneous dispersion. As Eosin Y is a hydrophilic fluorescent dye that emits in the green spectral region (∼540 – 560 nm) upon excitation (∼510 – 525 nm), it preferentially labels the hydrophilic matrix phase. Cleaned glass coverslips were treated with O2 plasma. The Eosin Y–containing solution was spin-coated onto the coverslips using a stepwise program (500 rpm for 5 s, 2000 rpm for 60 s, and 4000 rpm for 30 s). For UV-treated samples, the coated films were subsequently irradiated with UV light for 30 min. To maintain the hydrated state during imaging, a droplet of deionized water was placed on the film surface and covered with another coverslip. Imaging was carried out using a laser scanning confocal microscope (LSM980, ZEISS). Fluorescence was excited at 514 nm, and emission was collected in the range of 540–600 nm.

**X-ray photoelectron spectroscopy (XPS)**

Fully dried films were measured for XPS (K-alpha, Thermo VG Scientific) with a monochromic Al K-alpha source under a high vacuum condition of 10−9 Torr. Samples were prepared by spin-coating at 2000 rpm for 60 s with a ramp rate of 10.

**Scotch Tape Tests**

For Scotch tape tests, 1-inch × 1-inch PDMS substrates with a thickness of 500 µm were prepared by mixing the base and curing agent of Sylgard 184 (Dow Inc.) at a 20:1 ratio, followed by thermal curing onto the FTS-treated glass. The cured PDMS substrates were then transferred onto glass slides for the subsequent spin-coating process. After plasma treatment, each solution pure PEDOT:PSS, CS-mixed PEDOT:PSS, and PAS-mixed PEDOT:PSS (2.6 wt% of PAS) solution was spin-coated onto the substrates at 2000 rpm for 60 s with a ramp rate of 10. CPH_Pure_ and CPH_CS_ films were immersed in DMSO for 10 s and then annealed at 100 °C for 1 h. For CPH_PAS_ films, UV treatment (365 nm) was performed for 1 h prior to DMSO immersion and thermal annealing under the same conditions.

**Adhesion Characterization**

Adhesion tests were performed with a universal testing machine (UTM, Instron 68SC-1). For preparing PAS-mixed conductive polymer solution, 2.6 wt% of PAS was added to PEDOT:PSS solution. For preparing CPHCS, 0.4 wt% (6 mM) of Triton X-100 was added to PEDOT:PSS solution, which is the same molar concentration with PAS 2.6 wt%.

For lap-shear tests, one side of a slide glass (75 mm × 25 mm × 1 mm, MARIENFELD) was coated with PDMS (Sylgard 184, base: curing agent = 20:1 by weight) to a size of 15 mm × 25 mm and a thickness of 150 µm. The coated samples were left at room temperature for over 2 h to allow leveling, followed by complete curing at 50°C for 6 h. To prepare each CPH sample, 300 µL of the above conductive polymer solution was drop-casted onto the PDMS (same size as the PDMS layer) and dried overnight in a fume hood to obtain approximately 40 µm-thick CPH. After complete drying, the substrate was flipped over and irradiated with 365 nm UV light for 30 min from the transparent substrate side. The prepared samples were then immersed in deionized (DI) water for more than 2 h to achieve a fully swollen state. A nylon membrane filter (pore size = 0.45 µm, SciLab) was employed as backing layer to ensure strong adhesion to PEDOT:PSS, allowing delamination to occur specifically at the PDMS-CPH interface during testing without unwanted deformation, while also permitting water permeation [1, 2]. To reinforce the adhesion between the filter and PEDOT:PSS, the nylon filter was placed on the PEDOT:PSS film, hydrated for 1 h, and pressed at 50°C for 30 min using gentle force with hot pressing. The slide glass with PDMS attached via VHB tape was clamped on one gripper, while a nylon film was bonded on the other gripper with double-sided tape. After clamping, a sufficient amount of water was applied to the nylon filter side using a pipet, left for 15 min until fully hydrated. The lap-shear tests were conducted at a speed of 50 µm/s. Lap-shear strength is the maximum shear stress at the moment of failure.

For 90° peeling tests, each CPH sample was prepared by drop-casting the conductive polymer solution into a 2 mm-thick PDMS mold (10 mm × 15 mm) placed on a PDMS substrate with a thickness of 150 µm to obtain approximately 40 µm-thick CPH. After complete drying, the substrate was flipped over and irradiated with 365 nm UV light for 30 min from the transparent substrate side. The prepared samples were then immersed in deionized (DI) water for more than 2 h to achieve fully swollen state. The nylon filter was placed on the PEDOT:PSS film, hydrated for 1 h, and pressed at 50°C for 30 min using gentle force with hot pressing. After applying double-sided adhesive (VHB tape 4910, 3 M) to both the movable planar stage and the grippers, the nylon filter side was fixed to the stage, and the PDMS was clamped to the gripper. After being fully hydrated through nylon filter for 15 min, the PDMS was pulled upward at a rate of 50 µm/s for testing while maintaining a 90° angle between the PDMS and the filter. The peel-off strength was obtained by dividing the force by the width of the sample.

**Mechanical characterization (cyclic test, tensile test)**

Cyclic and tensile tests were performed using a universal testing machine (UTM, Instron 68SC-1) equipped with a 50 N load cell. All measurements were conducted at room temperature. For mechanical testing, film samples were cut into rectangular strips with a gauge length of 20 mm and a width of 2 mm. The initial thickness of each specimen was measured using a digital micrometer prior to testing. Samples were mounted between double-sided tape–attached grips to minimize slippage during testing.

For cyclic tensile tests, samples were stretched to a predetermined strain (20%) at a constant crosshead speed of 100% min−1 and subsequently unloaded to zero force. This loading–unloading cycle was repeated for 10 cycles. Stress–strain curves were recorded for each cycle.

For monotonic tensile tests, samples were stretched at a constant crosshead speed of 100% min−1 until failure. Engineering stress was calculated by dividing the applied force by the initial cross-sectional area, and tensile strain was defined as the ratio of extension to the initial length.

**Rheological characterization (frequency sweep, amplitude sweep)**

For rheological tests, samples were prepared as follows. A PEDOT:PSS–PAS mixed solution (or pure PEDOT:PSS solution) was cast into a 1.5 mm-thick PDMS mold and dried overnight to obtain dry films. For UV-treated CPH_PAS_ samples, both sides of the films were irradiated with 365 nm UV light for 30 min each. The films were then dipped once in DMSO, followed by annealing at 100°C for 2 h. Except for measurements conducted in the dry state, samples were immersed in deionized water for at least 30 min prior to testing to allow sufficient swelling.

Rheological measurements were performed using a rheometer (Anton Paar, MCR 302) equipped with an 8 mm parallel-plate geometry. All measurements were conducted at 25°C. The normal force was set to 0.25 N. Amplitude sweep measurements were carried out at a fixed angular frequency of 1 rad s−1 over a strain range of 0.01–100%. The storage modulus (G′) and loss modulus (G″) were recorded as a function of applied strain. Frequency sweep measurements were performed within the linear viscoelastic region (LVR) at a constant strain, as indicated in the corresponding figure, over an angular frequency range of 0.1–100 rad s^−1^. The storage modulus (G′) and loss modulus (G″) were recorded as a function of frequency. During rheological measurements of swollen hydrogel samples, the specimen was surrounded with a sufficient amount of deionized water to prevent dehydration throughout the test.

**Electrical Characterization (4-point probe, stretcher, EIS)**

For preparing PAS-mixed conductive polymer solution, 1.3 wt% of PAS was added to PEDOT:PSS solution. For preparing CS-mixed conducting polymer solution, 0.2 wt% (3 mM) of Triton X-100 was added, which is same molar concentration a PAS 1.3 wt%. For conductivity measurements, each conducting polymer solution (Pure, CS, PAS) is spin-coated with 3000 rpm for 60 s with ramp 10 s, forming a CPH with a thickness of approximately 150 nm and an area of 1 in × 1 in on a glass substrate. The thickness of each sample was measured with surface profiler (ET200, Kosaka Laboratory Ltd.). Sheet resistance of each sample was measured with a 4-point probe (M4P-205 System, MS TECH) connected to a source meter (2400 SourceMeter, Keithley). Conductivity was then calculated by the following equation: 𝜎 = $\frac{1}{R_{s}\cdot t}$, where R_s_ and t are the sheet resistance and thickness of CPH. Sheet resistance remained below 100 Ω·sq^−1^ for films thicker than 100 nm, indicating negligible thickness dependence in electrical performance (Figure S37). The measurement was conducted at 25 ± 3°C with a relative humidity of 50% ± 10%. For wet stability characterization of electrical conductivity, each sample was stabilized in D.I. water for 2 h.

Electrochemical impedance measurement of CPH_PAS_ and gold were performed by using a electrochemical impedance spectroscopy (VSP-300, BioLogic) in phosphate-buffered saline (PBS) with a platinum electrode as the counter electrode and Ag/AgCl as the reference electrode. Samples were prepared in the same manner as described for the conductivity measurements. The exposed area of each film in PBS was kept at 1 inch × 1 inch. Potentiostat electrochemical impedance spectroscopy (PEIS) was done with an amplitude of 10 mV, measured from 0.1 Hz to 1 MHz. For wet stability characterization of electrochemical property, all samples were stabilized by soaking in D.I. water for more than 2 days, and the 1-week, 2-week, 3-week, and 4-week data correspond to Day 7, Day 14, Day 21, and Day 28, respectively.

For electromechanical characterization, measuring the resistance of CPH_PAS_ when stretching, rectangular samples (10 mm × 1 mm) with square-shaped contact pads (3 mm × 3 mm) at the left and right sides of the rectangle were fabricated by a screen printing process. CPH_PAS_ ink (PEDOT:PSS solute: PAS = 1:1 or 1:2 weight ratio) was fabricated with reference to the following paper [3]: 0.16 g of PAS was dissolved in 2.67 mL of aqueous DMSO solution (water:DMSO  =  85:15 v/v) and stirred overnight. The pure PEDOT:PSS solution was frozen in liquid nitrogen bath for 10 min. The frozen PEDOT:PSS was lyophilized for 72 h to remove water and leave only solid PEDOT:PSS. 0.08 g of lyophilized PEDOT:PSS (for 1:1, 0.04 g for 1:2 weight ratio of PEDOT:PSS-to-PAS) were dissolved in mixture of above PAS solution, followed by grinding and overnight stirring. Masks for screen printing were made by cutting a polyethylene terephthalate (PET) film with a CO2 laser cutter (Trotec). After the CPH_PAS_ was printed, the substrate was flipped over and irradiated with 365 nm UV light for 30 min toward the transparent substrate-side. The samples were immersed in DMSO, annealed at 100°C for 1 h, and then soaked in D.I. water for washing and swelling. The prepared samples were mounted onto an electric motor using double-sided tape, and electrical pads were connected to external copper tapes with liquid metal(EGaIn) to minimize contact resistance at the pad–tape interface. The contact area was further fixed by encapsulating the pads with Scotch tape. Tensile tests were conducted at a strain rate of 100%/min using a bending and stretching machine system (SnM), while the electrical resistance was simultaneously recorded with a source meter (Keithley 2400).

**EMG Signal Measurements**

An electrophysiology data acquisition system (INTAN 128ch RHS Stim/Recording controller, Intan Technologies, USA) and its bundled software (Intan RHX) served as the data acquisition platform. The RHS2116 amplifier chip collected EMG with 20 kHz sampling rate. Each electrode is connected to reference and active electrode respectively. To eliminate stimulation artifacts, the “Amp Settle” feature was enabled; pre stim amp settle: 0 ms, post stim amp settle: 1 ms. Collected data were processed using a narrow-band 60 Hz notch filter (59.5 to 60.5 Hz) and band-pass filter (0.1 to 300 Hz) constructed in MATLAB ranging from 1 to 500 Hz, and RMS processing. Electrical pulse train (biphasic, 500 µA, 333µs and 1 Hz) yielded series of EMG signals and their averaged amplitude were used to evaluate the functional muscle activity. At least 10 peaks of EMG were used to determine muscular activity by the trigger technique.

**Animal Study and Experimental Design**

The animal care was followed by the National Institute of Health guidelines on the care and use of laboratory animals, and the handling of animal and experimental procedures were approved by the Institutional Animal Care and Use Committee, KIST (KIST-IACUC-2025-030). The experiments utilized male C57BL/6 mice that were 5 weeks old at the time of start animal study. To assess biocompatibility, PDMS (referred to as sham) and CPH_PAS_ on PDMS substrates were implanted into the muscle, and tissue responses were evaluated at 1- and 4-weeks post-implantation. Muscle tissues surrounding the implantation site of the sensor device were harvested for histological analysis (The surgical procedure and the extracted muscle area used for cell staining are illustrated in detail in Figure S20). To assess device functionality with EMG recording, murine brain tumor model was established using stereotactic injection stage with GL261/luciferase murine brain cell line. The formation of tumors was confirmed via bioluminescence imaging prior to EMG recording. To assess long-term stability and functionality of sensor device, device was implanted in healthy mice and its performance was evaluated at initial measurement (Week 1) and 4 weeks post-implantation.

**Biochemical Evaluation**

To evaluate the in vivo safety profile, mice (n = 3 per group) were implanted with PDMS (referred to as Sham), CPH_CS_ on PDMS (referred to as CPH_CS_) or CPH_PAS_ on PDMS (referred to as CPH_PAS_). At day 1 and 1 week after implantation, whole blood and serum were collected from mice, and complete blood count (CBC) and biochemical analysis were conducted at an animal diagnostic testing center (DK Korea Biotech, Seoul, Korea).

**Histological and Immunohistochemical Analyses**

After completion of animal experiment, the muscles or brains were harvested and immediately fixed in 10% neutral buffered formalin (NBF) for 24 h. Following fixation, the tissues were cryoprotected by sequential immersion in 10% and 30% sucrose solutions. The samples were then embedded in optimal cutting temperature (OCT) compound, and sectioned at a thickness of 5 µm for subsequent analyses. The tissue sections were air-dried at room temperature and rehydrated in PBS. H&E staining was performed using a commercial staining kit (ab245880, Abcam), and Masson's trichrome staining was conducted using a trichrome stain kit (KH07007, BQC). Stained sections were imaged using an Olympus IX70 fluorescence microscope. For the immunofluorescence staining, the tissue sections underwent antigen retrieval using a universal HIER antigen retrieval reagent (ab208572, Abcam). The sections were blocked with 5% bovine serum albumin in 1X PBS at room temperature for 60 min. The sections were incubated for 16 h at 4°C with primary antibody: NeuN (targeting neuronal nuclear, 1: 500, CST24307, Cell Signaling Technology), NF-H (targeting neurofilament, 1:500, NB500-416, Novus Biologicals). After washing with 1X PBS, the sections were incubated in a secondary antibody: Donkey Anti-Rabbit IgG H&L (Alexa Fluor 555) (Ab150074, 1:200, Abcam) and Goat Anti-Mouse IgG H&L (Alexa Fluor 488) (Ab150113, 1: 200, Abcam). This incubation was kept away from light and lasted for 1 h at room temperature, followed by washing with 1 X PBS and 10 mM CuSO4/50 mM NH4Cl solution. And the sections were rinsed with distilled water. The sections were sealed with mounting solution (ProLong Diamond Antifade Mountant with DAPI, Invitrogen) and coverslips and stored at room temperature without exposure to light. Sections were then imaged using confocal microscope Olympus FV3000. Co-localization analysis between DAPI and NeuN signals was performed using FIJI software (ImageJ) with the JACoP (Just Another Co-localization Plugin) plugin. Manders’ coefficients (M1 and M2) were calculated to quantify the extent of co-localization.

**Statistical Analysis**

Statistical analyses were performed in GraphPad Prism 8. Statistical analyses were assessed with an unpaired student's t test. Data are reported as mean ± SEM. The p-values of * p < 0.05, ** p < 0.01, and *** p < 0.001 were considered statistically significant.

Figure S1. Synthesis scheme of photoactivable surfactant (PAS). The synthetic scheme is divided into the following two steps, each described as follows. Step 1: Synthesis of BP-PDMS-OH, and Step 2: Synthesis of BP-PDMS-PEG (PAS).

Figure S2. 1H NMR spectrum of synthetic product. (a) ^1^H NMR spectrum of BP-PDMS-OH after column chromatography. (b) ^1^H NMR spectrum of BP-PDMS-PEG after column chromatography.

Figure S3. Bubble formation during mixing of PAS and PEDOT:PSS solution by surfactant-like structure of PAS. Unlike the pure condition in (a), the addition of surfactant in (b) leads to the formation of denser and smaller bubbles because the surfactant reduces the surface tension of water, thereby lowering the interfacial energy required for bubble formation and decreasing the critical pressure for bubble nucleation. Consequently, as shown in (c), smaller bubbles are generated under the same conditions[4]. Therefore, the condition in (d) with PAS addition resembles that in (b) with CS, as both lead to the formation of smaller and denser bubbles. This highlights that our PAS functions in a similar manner to a surfactant, like CS.

Figure S4. Comparison of coating uniformity among CPH_Pure_, CPH_CS_, and CPH_PAS_ samples. Photographic images showing coating uniformity of different CPH formulations. CPH_Pure_ exhibits poor coating uniformity, whereas both CPH_CS_ and CPH_PAS_ demonstrate good coating uniformity over the substrate surface.

Figure S5. Comparison of adhesion of CPH_Pure_ and CPH_CS_. (a) Photograph of the delamination between PDMS substrate and CPH_CS_. (b) 90° peel-off test confirming weak adhesion of CPH_Pure_ and CPH_CS_ to hydrophobic PDMS substrates.

Figure S6. ^13^C NMR spectrum of the reaction product between benzophenone and PDMS base (Sylgard^TM^ 184) before and after UV irradiation. After UV exposure, a new ¹³C NMR peak appears at 84 ppm, suggesting the formation of a covalent bond between the photoactivable anchor and the PDMS base. To further characterize the carbon environment, DEPT-135 spectra were acquired. In this spectrum, CH and CH₃ carbons appear as positive signals, CH₂ carbons as negative signals, and quaternary carbons yield no signal. The absence of a DEPT-135 signal at 84 ppm confirms that the newly formed peak corresponds to a quaternary carbon, supporting the covalent attachment of the benzophenone moiety to the PDMS backbone.

Figure S7. Electrical resistance change of CPH_PAS_ and CPH_CS_ samples before and after tape peeling. (a) Schematic illustration of the tape tests with resistance measurement. Films are pressed onto substrates using a Scotch tape at 1 N force, and changes in electrical resistance (*R*) are subsequently measured. (b) Normalized resistance change (Δ*R*/*R*₀) after tape peeling (n = 4). CPH_PAS_ samples exhibit negligible changes in resistance, indicating robust adhesion and stable conductivity. In contrast, CPH_CS_ samples become insulating, showing a drastic increase in resistance due to significant delamination.

Figure S8. Screen printing process and screen-printed CPH_PAS_. (a) Schematic representation of the fabrication process: PAS is added to a DMSO solution (15 v%) and stirred overnight, followed by addition of the PAS solution to freeze-dried PEDOT:PSS and grinding. The resulting mixture is screen-printed through an 80 µm-thick PET mask, then flipped toward the PDMS side and exposed to 365 nm UV light for 1 hour. (b) Photograph shows that CPH_PAS_ maintains intact adhesion to PDMS when agitated in water and soaking in water over 7 days. (c) Printing mask design and optical microscopy image of the screen-printed CPH_PAS_ pattern with a line width of 200 µm and a thickness of 100 µm.

Figure S9. Swelling ratio and water content of CPH_Pure_ and CPH_PAS_. Swelling ratio and water content of CPH_Pure_ and CPH_PAS_. It was observed that increasing the PAS concentration effectively suppressed the swelling behavior. Swelling ratio is calculated by (W_s_-W_d_)/W_d_ ×100 (%), W_s_ is a weight of fully swollen hydrogel, and W_d_ is the initial weight of dried film before swelling (n = 3). Water content is calculated by (W_s_-W_d_)/W_s_×100 (%). The measured values were obtained by calculating the average of three samples (n = 3).

Figure S10. 90° peel-off test of PEDOT:PSS films containing various concentrations of PAS additive. With increasing PAS content, the films exhibit enhanced adhesion properties. The curve for 2.6 wt% of CPH_PAS_ shows the highest strength, highlighting the role of PAS in interfacial adhesion of CPH and hydrophobic substrates.

Figure S11. X-ray photoelectron spectroscopy (XPS) analysis of CPH_Pure_ and CPH_PAS_ films. S 2p spectra of CPH_Pure_ (black circles) and CPH_PAS_ (blue circles) samples normalized by PEDOT peak, showing distinct height of PSS peaks. The CPH_PAS_ exhibits a 27.5% reduction in the integral of PSS peak compared to the CPH_Pure_ sample, indicating that the PEG moiety of PAS removes the insulating PSS shell from PEDOT via Coulombic interactions, improving electrical conduction.

Figure S12. Long-term wet stability of CPH_PAS_. Photographic images of CPH_PAS_ immersed in deionized water over 33 days. The square-shaped films retain their shape, integrity, and adhesion to the substrate without observable delamination or dissolution, indicating excellent long-term aqueous stability. Images were taken at Day 0, 7, 14, 21, 29, and 33.

Figure S13. Wet stability of CPH_PAS_ films compared with CPH_CS_. Photographs showing the water resistance of CPH_PAS_ compared to a CPH_CS_. (a) While the CPH_CS_ delaminates and disperses in water after 30 minutes, (b) the CPH_PAS_ film remains intact and adherent to the substrate.

Figure S14. Electrochemical impedance of CPH_PAS_ films. (a) Comparison of impedance spectra between bare gold electrodes and CPHPAS on gold. The CPHPAS shows reduced impedance, particularly in the low-frequency region, indicating improved interfacial properties. (b) Electrochemical characteristics of CPH_PAS_ under aqueous conditions over 4 days. The spectra show a gradual increase in impedance, which stabilizes after 2 days. This trend is attributed to the initial stabilization process, during which residual PEDOT:PSS is leached out.

Figure S15. Stretching tests of CPH_GOPS_. (A) Stress-strain curves of 3-glycidoxypropyltrimethoxysilane (GOPS)-mixed CPH (CPH_GOPS_) with different concentrations. Each sample was prepared following the references [5]. All samples containing GOPS exhibited brittleness, with electrical connectivity lost at strains below 20%. (B) Photograph of the GOPS 1 v/v% sample. Cracks are formed even at low strains, leading to immediate fracture. (C) Photograph of the GOPS 10 v/v% sample. The hydrogel becomes even more brittle and fractures into small fragments.

**Figure S16. Electrical and mechanical test of CPH_GOPS_.** (**a**) 90° peel test results of CPH_GOPS_ samples with varying concentrations. All samples were prepared with the same dimensions as those used in the PAS adhesion measurements and were tested under hydrated conditions with continuous water supply through a nylon filter. (**b**) Electrical conductivity at different GOPS concentrations. The conductivity decreases sharply when the GOPS content exceeds 5 v/v%. Based on the results in (**a**) and (**b**), 5 v/v% GOPS was selected as the control composition, as it provides sufficient conductivity while maintaining adhesion strength. (**c**) Impedance spectra of CPH_Pure_, CPH_GOPS_, and CPH_PAS_. All samples were prepared as 20 μm-thick dry bulk films and immersed in PBS for at least 30 min prior to measurement to ensure sufficient swelling. Consistent with previous reports [6, 7], CPH_GOPS_ exhibits limited swelling, which likely restricts ionic penetration and results in higher impedance.

**Z Height**

**a**

**Phase**

**b**

Figure S17. Atomic force microscope (AFM) images describing the bi-continuous structure driven by percolation of PAS and UV activation. (a) Z-height image and (b) phase image of patterned CPH_PAS_ regions. Scale bar = 200 nm.

Figure S18. Hydrogel formation of pure PAS after UV irradiation. (a) Photographs of pure PAS hydrogel. Pure PAS forms a hydrogel after UV irradiation followed by swelling in deionized water. (b) Stress–strain curve of pure PAS hydrogel obtained at a tensile rate of 100%/min. (c) Frequency sweep measurements of pure PAS hydrogel before and after UV exposure at 1% shear strain. It reveals a clear shift in the crossover point, indicating restricted chain mobility. This change suggests the formation of effective crosslinks that limit polymer chain relaxation dynamics. The emergence of more solid-like viscoelastic behavior after UV treatment supports that PAS alone can form a crosslinked hydrogel network.

Figure S19. (a) Swelling tests and (b) swelling ratio of CPH_Pure_ and CPH_PAS_ samples with varying PAS concentrations prior to DMSO treatment. When CPH_PAS_ bulk film samples before DMSO treatment are immersed in water, they do not dissolve but instead swell and form soft hydrogels. Since DMSO is known to enhance the connectivity within PEDOT:PSS networks, the absence of DMSO treatment minimizes PEDOT: PSS inter-network reinforcement. Under this condition, the crosslinking contribution of PAS becomes more clearly distinguishable. Notably, while CPH_Pure_ dissolves immediately in water, CPH samples containing PAS above the critical threshold (PAS 2.6 wt% & 5.2 wt%) no longer dissolve after UV irradiation and instead exhibit swelling behavior. For clarity, DMSO-treated pure PEDOT:PSS can also form a hydrogel network [4]. In our standard CPH_PAS_ fabrication process, DMSO treatment is applied.

Figure S20. Amplitude sweep of CPH_PAS, 2.6wt%_ before and after UV irradiation. The result shows a clear shift in yield point, indicating the formation of additional effective crosslinks even in the presence of PEDOT:PSS. This suggests that UV-induced PAS crosslinking occurs within the composite system. Amplitude sweep was performed at room temperature with a constant frequency of 1 rad/s.

Figure S21. Frequency sweep of (a) CPH_Pure_, (b) CPH_PAS, 2.6wt%_, and (c) pure PAS before and after swelling. The resulting CPH_PAS_ exhibits an elastic plateau over a broad frequency range within the linear viscoelastic region, where G′ remains greater than G″, consistent with the behavior observed for CPH_Pure_ and pure PAS. This frequency-independent storage modulus is consistent with the presence of a crosslinked network of hydrogels. All frequency sweep tests were performed at room temperature with a constant shear strain of 0.01%.

Figure S22. Viscoelastic behavior of CPH_PAS_. (a) Tensile tests of CPH_Pure_, CPH_PAS,1.3wt% ,_ CPH_PAS,2.6wt%_ and pure PAS. (b) Cyclic tensile tests of CPH_PAS, 2.6 wt%_. Over 10 consecutive loading–unloading cycles without rest time, the sample repeatedly returned close to its original length, indicating the presence of network constraints capable of elastic recovery. (c) Amplitude sweep of CPH_Pure_, CPH_PAS, 2.6wt%_, and Pure PAS at constant frequency of 1 rad/s. This showed that the incorporation of PAS network increases the yield point, indicating enhanced resistance to structural failure. Taken these together, both the PAS network and the PEDOT:PSS network contribute to the overall gel structure, forming interpenetrating and bi-continuous domains. The progressive reduction in modulus and stress at a given strain across cycles indicates gradual internal network damage and energy dissipation, which we primarily attribute to scission of the more brittle PEDOT:PSS-dominated framework. Importantly, the retention of macroscopic elasticity despite partial damage to the PEDOT:PSS network implies that the PAS-derived network is also percolated throughout the structure, supporting a bi-continuous network architecture. In this configuration, the PEDOT:PSS framework behaves similarly to a primary, damage-prone network, while the PAS network serves as a softer, ductile load-redistributing domain. These sustained elasticity and double-network-like mechanical behavior support the bi-continuous network structure.

Figure S23. Photopatterning capability above a threshold concentration (*C*_th_). Optical microscope images of irradiated with 365 nm UV light through a photomask; (a) PAS 0.7 wt%, (b) PAS 1.3 wt%, (c) PAS 2.6 wt%, (d) PAS 5.2 wt%. Photolithographic process was only successful above a certain threshold concentration (*C*_th_ = 1.3 wt%), which indicates the formation of a concentration-dependent molecular network. ‘SNU’ refers to Seoul National University, and ‘DMDL’ refers to the authors' laboratory (Dynamic Materials Design Lab). The use of the ‘SNU’ logo in this figure is authorized by Seoul National University.

Figure S24. Schematic illustrations of concentration-dependent network formation up to threshold concentration(*C*_th_) with photoactivation. At the initial stage, PAS exists as dispersed hydrophobic PDMS-rich domain within the PEDOT:PSS aqueous matrix. Due to the intrinsic immiscibility between the PDMS block and water, together with the asymmetric block-volume ratio, the hydrophobic segments locally segregate to minimize unfavorable interactions, forming discrete domains within the continuous hydrophilic environment. (i) At low PAS concentration (C << *C*_th_), these hydrophobic domains remain spatially isolated with large spatial distances between them. (ii) As the PAS concentration increases, the number density of hydrophobic domains rises, leading to reduced inter-domain spacing. (iii) Once the system approaches the *C*_th_, the domains become sufficiently close to enable network formation upon activation, although they are not yet covalently linked. (iv) Upon UV irradiation, benzophenone (BP) moieties are activated and covalently anchor adjacent hydrophobic domains, while the accompanying photothermal effect enhances chain mobility and transient intermixing at domain interfaces, thereby facilitating their stabilization into a continuous percolated network. As a result, the film becomes sufficiently crosslinked to remain intact and swell upon water immersion, ultimately forming a robust hydrogel structure.

Figure S25. Dynamic light scattering (DLS) analysis of pure PEDOT:PSS, pure PAS, and mixed PEDOT:PSS & PAS samples. The hydrodynamic particle size distribution shows distinct profiles for each sample, indicating differences in particle size distribution characteristics upon mixing. Mixed sample was prepared by diluting 20 μL of the original solution (PAS 2.6 wt% of PEDOT:PSS) in 8 mL of D.I. water.

Figure S26. Small-angle X-ray scattering (SAXS) analysis for CPH_PAS_ varying PAS concentrations. (a) Solution SAXS data tested on hydrogel precursor solution. In the solution SAXS, the strong scattering from PEDOT:PSS dominated the SAXS profile, making it impossible to observe the assemblies formed by PAS. (b) Bulk film SAXS data tested on dried bulk film of CPH_PAS_. This suggests that at low PAS concentrations (PAS < 2 wt%), the system exhibits a sparse density and minimal interconnection similar to a dilute polymer solution (slope < 1), whereas above a certain threshold (PAS > 2 wt%), the particles become more connected, forming a denser and more compact network structure (slope > 1) [8, 9]. While PAS particles are individually dispersed at lower concentrations, they begin to aggregate and form a distinct network structure once the concentration surpasses the threshold.

Figure S27. Conductivity variation of CPH_PAS_ as a function of PAS concentration. Conductivity profile showing the transition between two dominant mechanisms: (1) Surfactant-driven effect at low PAS concentrations (*C*_PAS_ < *C*_th_), where PAS acts as a polar additive, inducing π–π stacking of PEDOT by disrupting the interaction between PEDOT and PSS and enhancing the electrical phase; (2) Percolation-driven effect at high PAS concentrations (*C*_PAS_ > *C*_th_), where percolated PAS assemblies cause phase separation between the mechanical and electrical phases, with the mechanical phase becoming dominant. The optimal conductivity is achieved at approximately *C*_PAS_ = 1.3 wt %.

Figure S28. Schematic illustration of the mouse surgical procedure and cell extraction process for biocompatibility evaluation. (a) Anesthesia for surgery, (b) Skin incision above the target muscle for implantation, (c) Device implantation, (d) Suturing.

Figure S29. Renal biocompatibility assessment of CPH composites. Serum biochemical analysis of kidney function by measuring creatinine (CREA) levels in control, sham, CPH_CS_, and CPH_PAS_ groups. Data are presented as mean ± standard deviation (n = 3), indicating no significant differences among groups, which confirms the renal biocompatibility of the composites.

Figure S30. Serum biochemical analysis and complete blood count analysis. (a) Level of blood urea nitrogen (BUN) and (b) counts of red blood cells (RBC), (c) platelet (PLT), (d) monocytes (MONO), (e) eosinophil (EOS), and (f) basophil (BASO) were measured each time points. Data are presented as mean ± SD (n = 3). In the figure (a) ~ (f), the dashed line represents the normal mouse reference range.

Figure S31. Histological analysis of muscle and skin tissues 1 week post-implantation of CPH_CS_ sample. Representative images of hematoxylin and eosin (H&E; upper) and TUNEL staining (lower) are shown for (a, c) muscle and (b, d) skin tissue. The orange dashed line indicates the location of the implant. Scale bar = 200 μm.

Figure S32. Histological analysis of skin tissue 1 week post-implantation. Representative images of hematoxylin and eosin (H&E; upper) and TUNEL staining (lower). To clearly visualize the tissue layers, tissues were sectioned longitudinally rather than in a cross-sectional (wide-face) orientation. The orange dashed line indicates the location of the implant. Scale bar = 200 μm.

Figure S33. Histological analysis of muscle tissue at 1- and 4-weeks post-implantation. Representative images of hematoxylin and eosin (H&E, left) and Masson's trichrome (MT, right) staining. Scale bar = 100 μm.

Figure S34. TUNEL staining image. TUNEL staining images of tissue sections. Representative TUNEL staining images of tissue samples from the control, sham, and CPH_PAS_ groups at 1 and 4 weeks post-implantation. Nuclei were counterstained with DAPI and appear as circular blue fluorescence. Apoptotic cells were labeled using a BrdU-Red TUNEL staining kit, with red fluorescence. No significant increase in apoptotic signals was observed in the CPH_PAS_ group compared to the control and sham groups, suggesting minimal apoptosis induced by the implanted material.

Figure S35. EMG recordings without electrical stimulation. (a) Hind paw pinch stimulation with blank recordings. Upon mechanical stimulation, clear EMG bursts were observed, whereas no such activity was detected in the blank condition. (b) Blank signals without pinching stimulation. The absence of comparable activity in the blank recordings indicates that the observed signals are not attributable to filtering artifacts. (c) Recorded EMG signals with pinching. We compared EMG recordings acquired under identical experimental and processing conditions without stimulation. Importantly, both datasets were processed using the same filtering parameters as described in the manuscript. The absence of comparable activity in the blank recordings indicates that the observed signals are not attributable to filtering artifacts. Taken together, these results demonstrate that the filtering strategy used in this study is sufficient to isolate physiologically relevant EMG signals and that the recorded activity cannot be explained by bandpass-filtered artifacts alone.

Figure S36. Comparison of representative EMG traces upon electrical stimulation and signal-to-noise ratio(SNR) between (a) CPH_PAS_ and (b) CPH_GOPS_ sensors. Compared to CPH_PAS_, CPH_GOPS_ exhibits a significantly higher noise floor and lower SNR. Consistent with the impedance results, the limited swelling of GOPS-crosslinked films likely restricts ionic transport and interfacial coupling, resulting in reduced EMG signal fidelity. SNR was calculated after bandpass filtering by obtaining the RMS values of the signal and noise floor across ten stimulation segments for each sample.

Figure S37. Sheet resistance depending on film thickness. Films thicker than 100 nm showed consistent resistance values under 100 Ω·sq^-1^, indicating that there is no significant difference in electrical performance across different thicknesses in our system. Since electrical contacts between films and probes are relatively unstable when the film thickness decreases to several tens of nanometers, thin films resulted in relatively higher resistance. However, the increase was not significant, indicating that the overall electrical performance remained comparable.

Table S1. Summarized differences from the previous studies in terms of adhesion, wet stability, stretchability and electronic device applications.

| **Ref.** | **Major concept** | **Substrates** | **Adhesion**  **mechanism** | **Adhesion**  **force &**  **Wet stability** | **Stretch**  **-ability** | **Key advances**  **or limitations** |
| --- | --- | --- | --- | --- | --- | --- |
| **This work** | Photoactivable surfactant additive | PDMS | Decreasing the interfacial surface energy with covalent immobilization | **36.7 kPa,**  **169 N m**^-1^  **30-day incubation in D.I. water** | **> 300 %** | Strong wet adhesion on hydrophobic PDMS substrate, simultaneously achieving high mechanical stretchability. |
| **[10]** | Self-adhesive conductive polymer (SACP) with supramolecular solvents induced hydrogen bonds interactions. | PI, PEEK, Al, Cu, PET, PTFE, PDMS | Hydrogen bonds, ionic interaction, and Van der Waals’ interactions with supramolecular plasticizer | **>100 N m^-1^** for PI, PEEK, and PET  **N/A**  (It’s a form of elastomer, rather than hydrogel) | **~ 760%** | (1) Weak adhesion for hydrophobic substrate (>30 N/m for PDMS)  (2) It’s a elastomeric composite, rather than hydrogel. |
| **[1]** | Substrate amine functionalization & Hydrophilic polymer adhesive layer coating | Glass, polyimide, PDMS, ITO-glass, Gold | Interpenetration through PU layer which is covalently bonded to substrate | **>120 kPa**  **Remained intact after 60 min of ultrasonica-tion** | **~ 22 %** | (1) Multi-step processing with substrate functionalization  (2) Resulting interface is not stretchable. |
| **[11]** | Fatigue-resistant conducting hydrogel adhesion by high crystallinity | Glass, steel, Pt, PET, ITO-glass | Nanocrystalline domains of PVA suppressed crack propagation | **~ 1 kJ m^-2^** (Interfacial toughness)  **14-day incubation** in PBS | **N/A** | Adhesion is demonstrated only on stiff substrates. |
| **[12]** | A mixture of two emulsions: an aqueous conducting polymer, and hydrophobic & rubbery chains (PEA) copolymerized with silane coupling agents. | PDMS | Interlinking the PEA chains to the PDMS substrate with silane groups | **>300 N m^-1^**  **N/A** | **~ 140 %** | (1) Instability of silanol groups  (2) Instability of the two polymers to form an emulsion without surfactant assistance |
| **[13]** | Modified surfaces with chemical anchoring groups, which form strong covalent bonds with the conductive hydrogel | PU, metals | Covalent bonding with substrates functionalized with silane groups | **~ 300 kJ m^-2^**  (Interfacial toughness)  **N/A** | **> 300 %** | (1) Multi-step processing with substrate functionali-zation  (2) Instability of silanol groups |
| **[14]** | Light-curing polymer (LcP) enabling photopatternable biphasic conducting polymer hydrogel | PET,  3M 300LSE, PDMS | Hydrogen bonding between oxygen-rich groups of substrates and amino groups of LcP | **N/A**  (Scratch tests were performed instead for thin films)  Stability over 10,000  cycles in cyclic voltammetry (CV) curves | **~ 50%** | Based on relatively weak physical bonding compared to covalent bonding |
| **[15]** | High temperature (>150 °C) for a short time (≈ 2 min) baking | Glass, Gold, Parylene-C, PDMS, SEBS | High temperature induced phase separation of PEDOT and PSS, leading to the formation of linked, PEDOT-rich insoluble domains. | **N/A**  **20 days** both in vitro and in vivo | **< 35%** | Simple process, but (1) adhesion force and (2) wet stability is poor then this work. |
| **[16]** | Transfer-printing PEDOT:PSS films from glass to flexible substrates and baking. | PET, PDMS Hydrogel | Transfer printing of surfactant-mixed PEDOT:PSS from glass onto flexible substrates was facilitated by the stronger interfacial adhesion between PEDOT:PSS and the hydrogel. | **N/A**  The current level decreased by **~20%** after **72 hours** | **N/A** | No reports have been made on (1) adhesion force and (2) stretchability |
| **[17]** | Vacuum-assisted impregnation of PEDOT:PSS is presented onto an electrospun polyurethane (PU) nanofiber mat | PU nanofiber mat | Facile vacuum-assisted assembly of PEDOT:PSS results in a convoluted interlock network on the PU | **N/A**  **N/A** | **207%** | No reports have been made on (1) adhesion force and (2) wet stability |

**Supplementary References**

[1] A. Inoue, H. Yuk, B. Lu, and X. Zhao, “Strong Adhesion of Wet Conducting Polymers on Diverse Substrates,” Science Advances 6, no. 12 (2020): aay5394, <https://doi.org/10.1126/sciadv.aay5394>.

[2] D. Won, H. Kim, J. Kim, et al., “Laser-induced Wet Stability and Adhesion of Pure Conducting Polymer Hydrogels,” Nature Electronics 7, no. 6 (2024): 475–486, <https://doi.org/10.1038/s41928-024-01161-9>.

[3] H. Yuk, B. Lu, S. Lin, et al., “3d Printing of Conducting Polymers,” Nature Communications 11 (2020): 1604, <https://doi.org/10.1038/s41467-020-15316-7>.

[4] Q. Xu, M. Nakajima, S. Ichikawa et al., “Effects of surfactant and electrolyte concentrations on bubble formation and stabilization,” *Journal of Colloid and Interface Science* 332, no. 1 (2009): 208-214.

[5] G. L.-Buenafe, J. A.-Cabrera, C. Marcuello, “Fabrication and Characterization of PEDOT:PSS-Based Microstructured Electrodes for In Vitro Cell Culture”, Advanced Materials Interfaces, 12, no. 20 (2025): 2500097.

[6] M. ElMahmoudy, S. Inal, A. Charrier, I. Uguz, G. G. Malliaras, and S. Sanaur, “Tailoring the Electrochemical and Mechanical Properties of PEDOT:PSS Films for Bioelectronics”, Macromolecular Materials and Engineering, 302, no. 5 (2017): 1600497.

[7] A. Håkansson, S. Han, S. Wang et al., “Effect of (3-glycidyloxypropyl)trimethoxysilane (GOPS) on the electrical properties of PEDOT:PSS films”, Journal of Polymer Science Part B: Polymer Physics, 55, no. 10 (2017): 814-820.

[8] P.-G. De Gennes, “Scaling concepts in polymer physics,” Cornell University Press, Ithaca, NY (1979)

[9] Y. Gombert, F. Roncoroni, A.S.-Ferrer, and N.D. Spencer, “The hierarchical bulk molecular structure of poly(acrylamide) hydrogels: beyond the fishing net,” *Soft Matter* 16, (2020): 9789-9798.

[10] P. Tan, H. Wang, F. Xiao et al., “Solution-processable, soft, self-adhesive, and conductive polymer composites for soft electronics,” Nature Communications 13 (2022): 358. https://doi.org/10.1038/s41467-022-28027-y

[11] Y. Xue, X. Chen, F. Wang, J. Lin, and J. Liu, “Mechanically-Compliant Bioelectronic Interfaces through Fatigue-Resistant Conducting Polymer Hydrogel Coating,” Advanced Materials 35, no. 40 (2023): 2304095. <https://doi.org/10.1002/adma.202304095>

[12] G. Zhang, Z. Chen, C. H. Ahn, and Z. Suo, "Conducting Polymer Coatings Prepared by Mixed Emulsions Are Highly Conductive and Stable in Water," Advanced Materials 36, no. 13 (2024): 2306960.

[13] Y. Shin, H.S. Lee, Y.J. Hong et al., “Low-impedance tissue-device interface using homogeneously conductive hydrogels chemically bonded to stretchable bioelectronics,” Science Advances 10, no. 12 (2024): eadi7724. https://doi.org/10.1126/sciadv.adi7724

[14] W. Wang, J. Liu, H. Li et al., “Photopatternable PEDOT:PSS Hydrogels for High-Resolution Photolithography,” Advanced Science 12, no. 19 (2025): 2414834. https://doi.org/10.1002/advs.202414834

[15] S. Doshi, et al., “Thermal Processing Creates Water-Stable PEDOT:PSS Films for Bioelectronics,” Advanced Materials 37, (2025): 2415827. https://doi.org/10.1002/adma.202415827

[16] S. Zhang, et al., “Hydrogel-Enabled Transfer-Printing of Conducting Polymer Films for Soft Organic Bioelectronics,” Advanced Functional Materials 30, (2020): 1906016. https://doi.org/10.1002/adfm.201906016

[17] N. Keshmiri, P. Sambyal, P. Najmi, A. S. Milani, and M. Arjmand, “Stretchable Water-Repellent PEDOT:PSS-Impregnated Polyurethane Nanofiber Mats for Electromagnetic Interference Shielding,” Small 21, no. 35 (2025): 2500723. https://doi.org/10.1002/smll.202500723
